# CircARID1A binds to IGF2BP3 in gastric cancer and promotes cancer proliferation by forming a circARID1A-IGF2BP3-SLC7A5 RNA–protein ternary complex

**DOI:** 10.1186/s13046-022-02466-3

**Published:** 2022-08-19

**Authors:** Qiang Ma, Feifei Yang, Bo Huang, Xiaojuan Pan, Wei Li, Ting Yu, Xiaolin Wang, Lingyu Ran, Kun Qian, Hui Li, Haiping Li, Yuying Liu, Ce Liang, Junwu Ren, Yuying Zhang, Shimin Wang, Bin Xiao

**Affiliations:** 1 College of Pharmacy, Chongqing Medical University, Chongqing, 400016 P.R. China; 2 Department of Clinical Laboratory, Affiliated Hospital of North Sichuan Medical College, Nanchong, 637000 P.R. China; 3 Department of Pharmacy, Southwest Hospital, Army Medical University, Chongqing, 400038 P.R. China; 4 Department of Clinical Laboratory, The 89th Hospital of The People’s Liberation Army, Weifang, 261000 P.R. China; 5 Department of Kidney, Southwest Hospital, Army Medical University, Chongqing, 400038 P.R. China; 6 Department of Gastrointestinal Surgery, The First Affiliated Hospital, Chongqing Medical University, Chongqing, 400016 P.R. China

**Keywords:** circARID1A, IGF2BP3, SLC7A5, RNA–protein ternary complex, Gastric cancer

## Abstract

**Background:**

Gastric cancer (GC) is one of the most common malignant tumors in China. Circular RNAs (circRNAs) are novel non-coding RNAs with important regulatory roles in cancer progression. IGF2BP3 has been found to play oncogenic roles in various cancers including GC, while the exact mechanism of IGF2BP3 is largely unknown.

**Methods:**

The expression of IGF2BP3 in GC was evaluated by Western Blot and bioinformatics analysis. CircRNA expression profiles were screened via IGF2BP3 RIP-seq in GC. Sanger sequencing, RNase R digestion, nucleo-plasmic separation and RNA-FISH assays were used to detect the existence and expression of circARID1A. RNA ISH assay was employed to test the expression of circARID1A in paraffin-embedded GC tissues. Moreover, the function of circARID1A on cellular proliferation was assessed by CCK-8, plate colony formation, EdU assays and GC xenograft mouse model in vivo. Furthermore, the location or binding of circARID1A, IGF2BP3 protein and SLC7A5 in GC was evaluated by RNA-FISH/IF or RNA pull-down assays.

**Results:**

We identified a novel circRNA, circARID1A, that can bind to IGF2BP3 protein. CircARID1A was significantly upregulated in GC tissues compared with noncancerous tissues and positively correlated with tumor length, tumor volume, and TNM stage. CircARID1A knockdown inhibited the proliferation of GC cells *in vitro* and *in vivo* and circARID1A played an important role in the oncogenic function of IGF2BP3. Mechanistically, circARID1A served as a scaffold to facilitate the interaction between IGF2BP3 and SLC7A5 mRNA, finally increasing SLC7A5 mRNA stability. Additionally, circARID1A was able to directly bind SLC7A5 mRNA through complementary base-pairing and then formed the circARID1A-IGF2BP3-SLC7A5 RNA–protein ternary complex and promoted the proliferation of GC via regulating AKT/mTOR pathway.

**Conclusions:**

Altogether, our data suggest that circARID1A is involved in the function of IGF2BP3 and GC proliferation, and the circARID1A-IGF2BP3-SLC7A5 axis has the potential to serve as a novel therapeutic target for GC.

**Supplementary Information:**

The online version contains supplementary material available at 10.1186/s13046-022-02466-3.

## Background

Gastric cancer (GC) is a global public health issue with over 1 million estimated new cases annually and there were 784 000 deaths from GC related diseases in 2018 [1]. In China, the morbidity and mortality for GC ranked second among all malignant diseases in 2015 [2]. Patients in the early disease stages can be cured by timely radical surgery [3]. For patients with unresectable advanced or recurrent gastric cancer, the clinical efficacy is limited with the standard first-line therapy of fluoropyrimidine- and platinum-based chemotherapies [3, 4]. Thus, there is an urgent need to uncover the underlying mechanisms of GC to enable the development of more effective therapeutic targets for GC.

RNA-binding proteins (RBPs) are essential players in RNA metabolism and regulate RNA splicing, transport, surveillance, decay and translation [5]. RBPs also participate in tumorigenesis and progression and are potential targets for tumor therapy [6–8]. The insulin like growth factor II mRNA binding protein 3 (IGF2BP3) is a mammalian IGF2 mRNA-binding protein family member and contains 2 RNA recognition motifs (RRM) in its N-terminus and 4 hnRNPK homology (KH) domains at the C-terminus. IGF2BP3 plays an oncogenic role in the tumorigenesis of certain tumors and is associated with tumor progression [9–13]. IGF2BP3 is elevated in GC and negatively associated with overall survival and recurrence-free survival [14]. Our previous research and that of others confirmed that IGF2BP3 functions as an oncogene that facilitates tumorigenesis, metastasis, and GC progression by interacting with mRNA and noncoding (nc) RNAs [15–17]. However, the mechanisms explaining the oncogenic role of IGF2BP3 in GC has not been fully clarified. In particular, identification of interactions of IGF2BP3 with ncRNAs provides a starting point to clarify its role in oncogenesis.

Circular RNAs (circRNAs) form a novel class of ncRNAs and are expressed during developmental process and in certain disease states [18–20]. CircRNAs are covalently closed single-stranded transcripts that are derived from pre-mRNA by back-splicing and lack 5′ G-caps and 3′ A tails. This class of RNAs possesses enhanced stability in the cell due to its inaccessibility to the action of RNA exo- and endonucleases. CircRNAs can act as miRNAs sponge, protein scaffolds, transporters, or decoys, translational template, and transcriptional regulators [21]. Interestingly, there is aberrant circRNA expression in GC and these molecules are linked to GC progression through interactions with miRNAs and proteins [22–24]. Whether circRNAs and IGF2BP3 interact in cancer or even under homeostatic conditions is largely unknown.

SLC7A5 (solute carrier family 7 member 5 or LAT1) is a transporter of large branched-chain and aromatic neutral amino acids [25, 26] and is highly expressed in breast cancer, non-Hodgkin's lymphoma and colorectal cancer and positively associated with poor prognoses [27–29]. Moreover, SLC7A5 can promote cancer proliferation via AKT/mTORC1 pathway activation [30]. Furthermore, SLC7A5 is a candidate molecular target for cancer therapeutics and inhibition of transport with JPH203 (Nanvuranlat) significantly suppressed the proliferation of estrogen deprivation-resistant (EDR) breast carcinoma cell lines [31]. Additionally, SLC7A5 expression is upregulated in GC and this was linked to GC disease progression and contributed to chemotherapeutic resistance in GC patients [32–34].

In the present study, we employed RIP-Seq to screen for circRNAs that bind to IGF2BP3 in SGC7901 cells and identified circARID1A (CircBase ID: hsa_circ_0008494) that was derived from the pre-mRNA of the ARID1A gene and was upregulated in GC and directly interacted with IGF2BP3. Functionally, circARID1A promoted the proliferation of GC and mechanistically elevated SLC7A5 expression by enhancing its stability via the formation of a circARID1A-IGF2BP3-SLC7A5 RNA–protein ternary complex in GC. This study provides a novel therapeutic target for the treatment of GC.

## Methods

### Human GC specimens

GC and the corresponding noncancerous tissue samples (21 pairs) were obtained from Southwest Hospital of the Army Medical University. All patients were diagnosed by histopathological examination and were without chemotherapy or radiotherapy prior to surgery. The specimens were collected immediately in EP tubes containing RNA Later (Thermo Scientific, Pittsburg, PA, USA) after surgical resection and stored at -80 °C. Clinical characteristics of GC patients were given in Supporting Information Table S1. Informed consent was obtained from patients for each sample and the study was approved by the Ethics Review Committee of Chongqing Medical University and Southwest Hospital of the Army Medical University.

### Cell lines

The GC-derived cell lines SGC7901, BGC823, and HGC-27 cells or normal gastric epithelial cell GES-1 were obtained from the Army Medical University (Chongqing, China). AGS and MKN-74 cells were purchased from Meisen (Hangzhou, China). All cells were cultured at 37 °C in a 5% CO_2_ atmosphere in DMEM or RPMI 1640 medium (Basal Media, Shanghai, China) supplemented with 10% fetal bovine serum (PAN-Biotech GmbH, Adenbach, Germany).

### Western blot

Total protein was extracted from cultured cells using RIPA lysis buffer containing protease inhibitors (Beyotime, Shanghai, China). Cell lysates were separated on SDS–polyacrylamide gels and electrotransferred onto polyvinylidene difluoride (PVDF) membranes that were incubated at 4 °C with primary antibodies in 5% nonfat milk in PBS containing Tween-20 (PBST). The antibodies used for this study were used at 1:1000 dilutions in primary antibody dilution buffer (Beyotime) and were as follows: GAPDH and β-actin (Beyotime), SLC7A5 (Proteintech, Chicago, USA), IGF2BP3 (Abcam, Cambridge, UK), mTOR, p-mTOR, AKT and p-AKT (Cell Signaling Technology, Beverly, MA, USA). Membranes were washed with PBST for three times and incubated with HRP-conjugated secondary antibodies for 1 h at room temperature. The blots were visualized using an ECL chemiluminescent reagent (Bioground, Chongqing, China).

### Gene knockdown and overexpression

SiRNAs targeting IGF2BP3, circARID1A, and SLC7A5 were synthesized by GenePharma (Shanghai, China) (Supporting Information Table S2). Briefly, cells that had been seeded into 6 well plates overnight were transfected with siRNAs using lipofectamine 2000 (Invitrogen, Carlsbad, CA, USA) according to the manufacturer’s instructions and incubated for 6 h. The medium was then replaced and cells were collected at indicated time points (see below).

For stable expression of IGF2BP3, pCDH-IGF2BP3 plasmids were constructed and co-packaged using a commercial lentivirus system that was assisted using pMD2.G, pCMV-VSV-G, and pRSV-Rev plasmids in 293T cells. SGC7901 and BGC823 cells were then infected with lentivirus-IGF2BP3 overexpression and stable cell lines were selected by the addition of puromycin. Transfection efficiency was confirmed by Western blot using IGF2BP3 antibodies (see above). For IGF2BP3 transient overexpression, the overall CDS region of IGF2BP3 was inserted into pcDNA3.1( +) for IGF2BP3 overexpression. The vector was transfected into GC cells using a commercial DNA transfection reagent (Neofect, Beijing, China).

### Cell Counting Kit-8 assay

Cells were seeded into 96-well plate at 5 × 10^4^/mL using 100 μL/well and incubated for the indicated times (see below). 10 μL of Cell Counting Kit-8 (CCK-8) (Biosharp, Hefei, China) reagent was added to each well and the plate was incubated at 37 °C for 2 h in a 5% CO_2_ atmosphere, the absorbance was measured at 450 nm using a microplate reader (Thermo).

### Plate colony formation assay

Cells were removed from monolayers using trypsin digestion and seeded into 12-well plates at 1 × 10^3^ cells per well. Two weeks after seeding, tumor spheres were fixed using 4% paraformaldehyde for 10 min and then stained with 1% crystal violet solution for 10 min at room temperature. The colony spheres were washed 3 × with water and then dried at room temperature. Plate images were captured using an Epson scanner (Suwa, Nagano Prefecture, Japan).

### EdU (5-ethynyl-2´-deoxyuridine) incorporation assay

GC cells were treated and seeded into 48-well plate at a density of 3 × 10^4^ per well. EdU incorporation utilized a Cell-Light EdU Apollo 567 In Vitro Kit (RiboBio, Guangzhou, China). Cells were treated and exposed to EdU solution (50 μM) for 2 h at 37 °C and then processed according to the manufacturer’s instructions. Cells were observed and photographed using a fluorescent inverted microscope (Olympus, Tokyo, Japan).

### RNA-binding protein immunoprecipitation (RIP) assay

RIP assays were performed according to the instructions of Magna RIP RNA-Binding Protein Immunoprecipitation Kit (Millipore, Burlington, MA, USA). Briefly, cells were lysed with RIP lysis buffer and incubated with antibodies specific for IGF2BP3 or rabbit IgG. Co-precipitated RNAs were purified for sequencing or cDNA synthesis and used as template for indicated gene expression detection by qRT-PCR with specific primers. Total RNA (Input) or antibody (Rabbit IgG) was used as references.

### Actinomycin D assay

For circARID1A stability evaluation, SGC7901 and BGC823 cells were seeded into 12 well plates and treated with actinomycin D (5 μg/mL) (Genview, Beijing, China) for 0, 3, 6, and 9 h. Cells were lysed in RNAiso Plus (Takara, Kyoto, Japan) for RNA extraction and qRT-PCR measurements. For mRNA stability assay, SGC7901 and BGC823 cells were seeded into 12 well plates and treated with si-circARID1A or si-IGF2BP3 and then treated with actinomycin D (20 μg/mL) or vehicle for 0, 3, 6, and 9 h. Cells were lysed in RNAiso Plus (Takara) for RNA extraction and further qRT-PCR detection.

### RNase R treatment

SGC7901 and BGC823 cells were seeded into 12 well plates and incubated overnight. Cells were used for total RNA extraction and a total of 5 µg RNA was incubated in the presence or absence of RNase R (6 U) (Lucigen, Middleton, WI, USA) at 37 °C for 10 min, followed by 85 °C for 5 s. After RNase R treatment, the expression of linear ARID1A and circARID1A were measured using qRT-PCR.

### Nuclear-cytoplasmic fractionation

Nuclear and cytoplasmic RNA in SGC7901 and BGC823 cells were isolated using the Paris Kit (Life Technologies, Gaithersburg, MD, USA) according to the manufacturer’s instructions. For circARID1A, ARID1A, and GAPDH, cDNA was synthesized with a PrimeScript RT Master Mix (Takara) and for snoU6 the cDNA was synthesized by stem-loop methods (RiboBio). Quantitative real-time PCR (qRT-PCR) analysis was executed using a SYBR Green Master Mix (Bioground). The 2^−ΔΔCt^ method was used to analyze the relative expression levels of genes in nuclear and cytoplasm [35].

### RNA fluorescence in situ hybridization (RNA-FISH)

Biotin-labeled probes or FAM-labeled probes were synthesized by Genepharma and used to visualize circARID1A or SLC7A5 in situ. Briefly, SGC7901 and BGC823 cells were seeded into μ-Slide 8-well chamber slide (Ibidi, Martinsried, Germany). The medium was removed 24 h later and cells were washed 2 × with PBS. Then cells were fixed with 4% paraformaldehyde for 10 min and treated with Triton X-100 (0.5%) at room temperature for 15 min. Cells were washed 2 × with PBS and incubated with denatured probes targeting circARID1A, SLC7A5, 18S rRNA or negative control and the slides were incubated at 37 °C overnight. The next day, nuclei were counterstained with DAPI and images were taken using a laser confocal microscope (Leica SP8, Wetzlar, Germany). The probe sequences for RNA-FISH are listed in Supporting Information Table S3.

### Total RNA extraction and quantitative real-time polymerase chain reaction

Total RNA was extracted with RNAiso Plus (Takara) following the manufacturer's instructions. RNA was quantified using a Nanophotometer (Implen, Munich, Germany) and cDNA was synthesized using a PrimeScript RT Master Mix (Takara). qRT-PCR was conducted using SYBR Green Master Mix (Bioground) with CFX connect Real-Time PCR System (BioRad, Hercules, CA, USA). Human *GAPDH* was used as an internal control for the relative expression of circRNAs and mRNAs. The 2^−ΔΔCt^ method was used to calculate the relative expression of indicated genes and primers are listed in Supporting Information Table S4.

### Pull-down assay with biotinylated circARID1A probe

RNA pull-down assay was performed using a Waals RNA Pull down Kit (Chongqing, China). Briefly, SGC7901 or BGC823 cells (1 × 10^7^) were harvested and lysed in IP lysis buffer on ice for 30 min. Avidin-labeled magnetic beads were incubated with biotin-labeled circARID1A probes or control probes (GenePharma) at 25 °C for 2 h to generate probe-coated beads. The cell lysates were incubated with circARID1A probe or control probe to pull-down circARID1A. The RNA complexes bound to the beads were purification using phenol/chloroform/isoamyl alcohol (25: 24: 1) for real-time PCR measurements. The protein complexes bound to the beads were denatured by heating at 100 °C for 10 min for Western blot analysis. The probe sequences for RNA pull-down are listed in Supporting Information Table S5.

### Immunofluorescence assay

GC cells grown on confocal dishes (Ibidi) were fixed using 4% paraformaldehyde for 10 min, and permeabilized with 0.1% Triton X-100 in PBS for 10 min. Cells were washed 2 × with PBS and blocked with 5% BSA for 30 min at 37 °C, and incubated with specific primary antibody at 4 °C overnight. The primary antibody was removed and cells were washed 3 × with PBS and incubated with corresponding secondary antibody for 30 min at 37 °C, followed by staining with DAPI. Fluorescent images were obtained using a Leica SP8 confocal microscope.

### Tissue microarray (TMA) and in situ hybridization (ISH)

TMA were produced from 180 paraffin-embedded GC or adjacent tissues by Outdo Biotech (Shanghai, China). The clinical pathological characteristics including gender, age, survival time, pathological grading, tumor volume, TNM stage, AJCC stage, the expression of PDL1 or CD8 were public data and provided by Outdo Biotech (Shanghai, China). ISH was employed to detect the expression of circARID1A in GC tissues. The probes targeting the junction of circARID1A were synthesized by Boster (Wuhan, China) and the ISH Kit was purchased from Boster. Briefly, TMA was dewaxed in xylene and rehydrated with 100, 95, 85 and 75% ethanol and digested with trypsin. The TMA were hybridized with specific digoxin-labeled circARID1A probes overnight. The specimens were incubated with biotin-conjugated anti-digoxin antibody (Roche, Basel, Switzerland), and incubated with avidin-conjugated peroxidase and stained with NBT/BCIP (Roche) and photographed. circARID1A expression was quantified and analyzed. The positive staining intensity (0, no expression; 1, mildly positive; 2, moderately positive; and 3, markedly positive) was multiplied by the percentage of positive staining cells (0, < 5%; 1, 6–25%; 2, 26–50%; 3, 51–75%; 4, > 75%) to calculate the score for ISH staining. The probe sequence is listed in Supporting Information Table S6.

### Tumor-bearing nude mice model construction

Lentivirus constructs containing an shRNA targeting circARID1A was packaged by Hanbio (Shanghai, China) with a virus titer of 10^8^/UI. SGC7901 cells were infected with lentivirus at a MOI = 50 using polybrene. The transfection efficiency was measured and proliferation of the SGC7901 stable circARID1A knockdown cells was detected. Briefly, 10 male BABL/C nude mice (Gempharmatech, Jiangsu, China) aged at 3–4 weeks were randomly divided into two groups. SGC7901 cells transfected with sh-NC or sh-circARID1A were cultured and digested with 0.25% trypsin. Cells were washed twice with cold PBS and diluted with PBS at a concentration of 3 × 10^7^/mL. 100 µL of cell suspension was subcutaneously implanted in the upper-right flank of nude mice. One week later, the length and width of the tumor tissues was measured every two days. The volume of the tumor size was calculated by the formula: volume (mm^3^) = length × width^2^ / 2. The experiment was terminated one month after xenograft mouse model construction and the animals were sacrificed and tumor weight was measured. The tumor tissues were collected for indicated gene expression evaluation and IHC staining.

For in vivo rescue assay of circARID1A and SLC7A5, HGC-27 cells were employed to construct xenograft tumor model in nude mice. Briefly, 3-week-old male BALB/C nude mice (Gempharmatech) were subcutaneously injected in the right flank with HGC-27 cells (1.5 × 10^6^) in 0.1 mL phosphate-buffered saline (PBS). When the tumor had grown to an appropriate volume, the tumor-bearing mice were randomly divided into three groups (n = 4). In vivo treatment with siRNA in the mouse models was performed as previously described [36]. Then, cholesterol-modified si-circARID1A or si-RNA control (GenePharma, 5 nmol/kg) with 5 μL in vivo transfection reagent (Entran-ster™-in vivo, Engreen, Beijing, China) was locally injected into the tumor mass once every 2 days for 2 weeks. Moreover, lentivirus containing SLC7A5 were also injected into the tumor mass at day 1 and day 3. The study was approved by the Ethics Review Committee of Chongqing Medical University.

### Immunohistochemistry (IHC) staining

IHC was performed on formalin-fixed, paraffin-embedded tissue sections as described previously [37]. The primary antibody used was Ki-67 (1:500, Servicebio, Wuhan, China). Tissue sections were incubated with primary antibody at 4 °C overnight and then incubated with secondary antibody. DAB complex was used as the chromogen and the nuclei were counterstained with hematoxylin. Three different visual fields were randomly selected for each slice and all images were scored as follows: proportion score: 0–4 (0, < 5%; 1, 6–25%; 2, 26–50%; 3, 51–75%; 4, > 75%); intensity score: 0, negative; 1, weak; 2, intermediate; 3, strong; total score = proportion score × intensity score.

### RNA-sequencing

SGC7901 cells were seeded to 6-well plate at a density of 5 × 10^5^ cells per well. Twenty-four hours later, cells were transfected with siRNAs targeting to circARID1A with the help of lipofectamine 2000 reagent (Invitrogen) according to the manufacturer’s instructions. Six hours later, the supernatant was replaced with fresh DMEM containing 10% fetal bovine serum. Cells were collected after 48 h for further RNA sequencing by Sinotech Genomics (Shanghai, China).

### Statistical analysis

Student’s t test or one-way analysis of variance (ANOVA) was used to compare the differences of continuous variable between two groups or among multiple groups. Kaplan–Meier analysis was employed for survival analysis, and the differences in the survival probabilities were estimated using the log-rank test. ISH score of circARID1A was categorical variable, and was analyzed by Kruskal–Wallis Test. The statistical analyses were performed using SPSS version 22.0 (IBM, Chicago, Ill, USA) and Prism 8.0 (GraphPad, San Diego, CA, USA). *P* < 0.05 was considered to indicate statistical significance.

## Results

### IGF2BP3 is elevated in gastric cancer and promotes the proliferation of GC *in vitro*

**Fig. 1:**
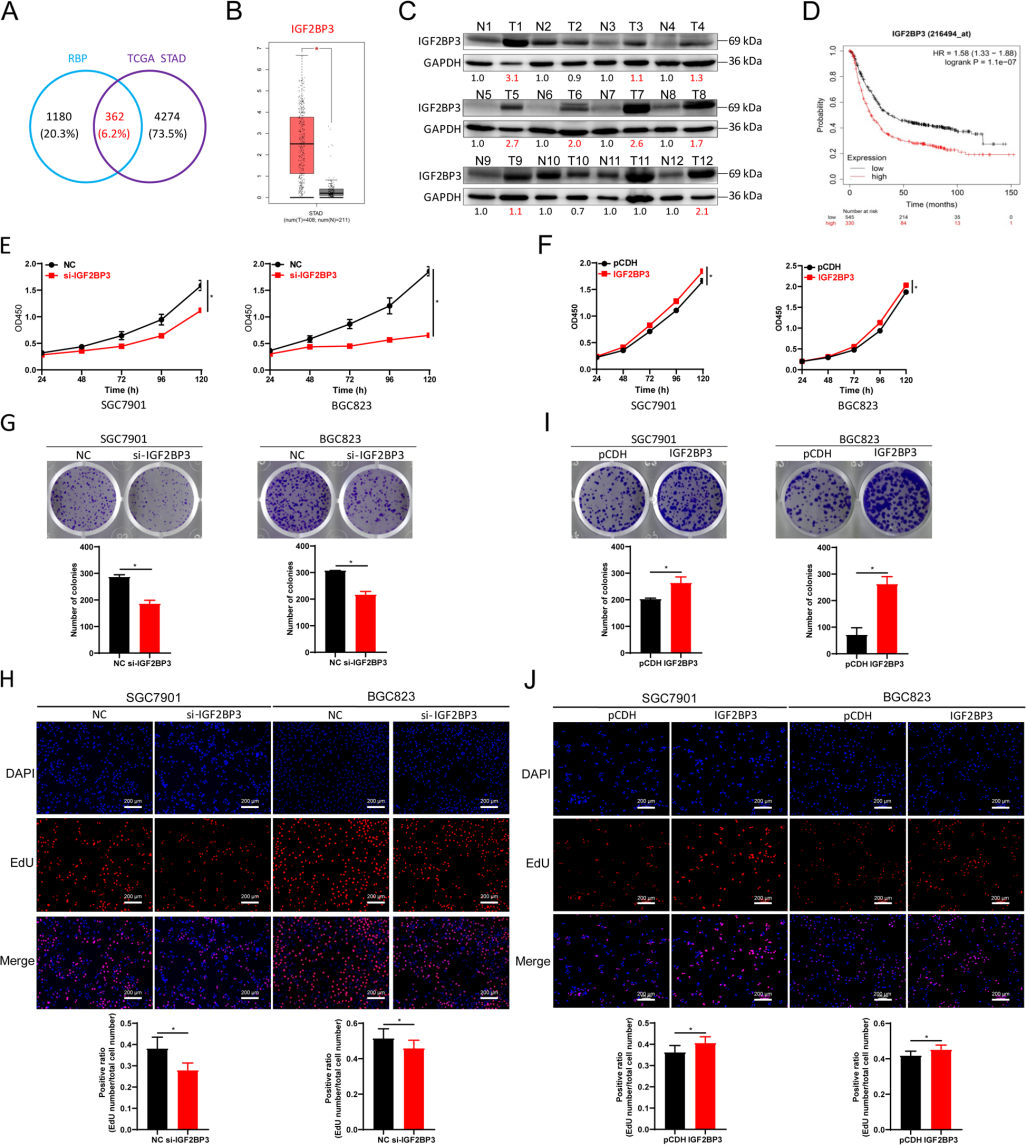
IGF2BP3 promotes the proliferation of GC. **A** 362 overlapping genes between RBP genes (1542) and differential expression genes of STAD from the TCGA database. **B** IGF2BP3 mRNA expression between GC tissues and adjacent tissues of STAD in the TCGA database. **C** IGF2BP3 protein expression in GC tissues. **D** Association between IGF2BP3 expression and the overall survival in GC patients from the Kaplan–Meier plotter database. **E**–**F** CCK-8 assay of SGC7901 and BGC823 cells following IGF2BP3 (**E**) knockdown or (**F**) stable overexpression. **G** Plate colony formation assay of SGC7901 and BGC823 cells following IGF2BP3 knockdown. **H** EdU assay of SGC7901 and BGC823 cells following IGF2BP3 knockdown. **I** Plate colony formation assay in SGC7901 and BGC823 cells that stably overexpressed IGF2BP3. **J** EdU assay in SGC7901 and BGC823 cells stably overexpressing IGF2BP3. The *P*-values were calculated using the two-tailed Student’s t test. *P < 0.05

RBPs play indispensable roles in RNA metabolism and are linked to tumorigenesis and metastasis of solid tumors and hematopoietic lymphatic system tumors. There are currently 1542 RBPs in humans [38] and overlapping analysis with differentially expressed genes of GC patients from the TCGA database indicated that there are 362 RBPs aberrantly expressed in GC (Fig. 1A, Supporting Information Tables S7-S8). Among these differentially expressed genes, IGF2BP3 is one of the most upregulated genes in GC (Supporting Information Fig. S1A, Fig. 1B). We used 12 paired GC and peritumor tissues to validate IGF2BP3 expression in GC and found that the IGF2BP3 protein was significantly upregulated in 75% (9/12) of the GC tissues (Fig. 1C). Furthermore, expression analysis for IGF2BP3 in the Human Protein Atlas (https://www.proteinatlas.org/) consistently confirmed that IGF2BP3 was up-regulated in GC compared with normal gastric mucosa (Supporting Information Fig. S1B). IGF2BP3 was also significantly up-regulated in the GC cell lines SGC7901, BGC823, AGS and MKN74 cells compared with normal gastric epithelial cell line GES-1 (Supporting Information Fig. S1C). Additionally, worse overall survival was observed in GC patients with higher IGF2BP3 expression from Kaplan–Meier Plotter database [39] (Fig. 1D).

To further uncover the role of IGF2BP3 in GC, we measured proliferation of GC cell lines following IGF2BP3 knockdown or overexpression. Firstly, we designed two siRNAs targeting IGF2BP3 and evaluated the interference efficiency and function in GC cells, then we chose si-IGF2BP3-1 with better inhibition efficiency of proliferation (hereafter called si-IGF2BP3) for subsequent study (Supporting Information Fig. S2). Cell viability was significantly inhibited in SGC7901 and BGC823 cells after IGF2BP3 knockdown (Supporting Information Fig. S3A and Fig. 1E) but increased after IGF2BP3 overexpression (Supporting Information Fig. S3B and Fig. 1F). Proliferation of SGC7901 and BGC823 cells were further evaluated by plate colony formation and EdU assays. The results indicated that knockdown of IGF2BP3 suppressed the proliferation of SGC7901 and BCG823 cells (Fig. 1G-H). As expected, ectopic expression of IGF2BP3 facilitated the proliferation of GC cells (Fig. 1I-J). Altogether, these results demonstrated that IGF2BP3 was an RBP with significantly increased expression in GC and it promoted GC cell proliferation.

### CircARID1A interacts with IGF2BP3 protein in GC

As an RNA binding protein, IGF2BP3 was reported to bind mRNA and ncRNAs and regulate target gene functions [40, 41]. We therefore identify circRNAs that bound IGF2BP3. We identified > 50 circRNAs that interacted with IGF2BP3 protein and the top 20 circRNAs were shown (Supporting Information Fig. S4A and Table S9), which showed circELK4 displayed the greatest level of binding. However, the expression of circELK4 in GC tissues and adjacent tissues was not significant difference in our cohort (Supporting Information Fig. S4B), we then focused on the second most abundant binding circRNA, circARID1A, and further evaluated the interactions between circARID1A and IGF2BP3 protein.

**Fig. 2:**
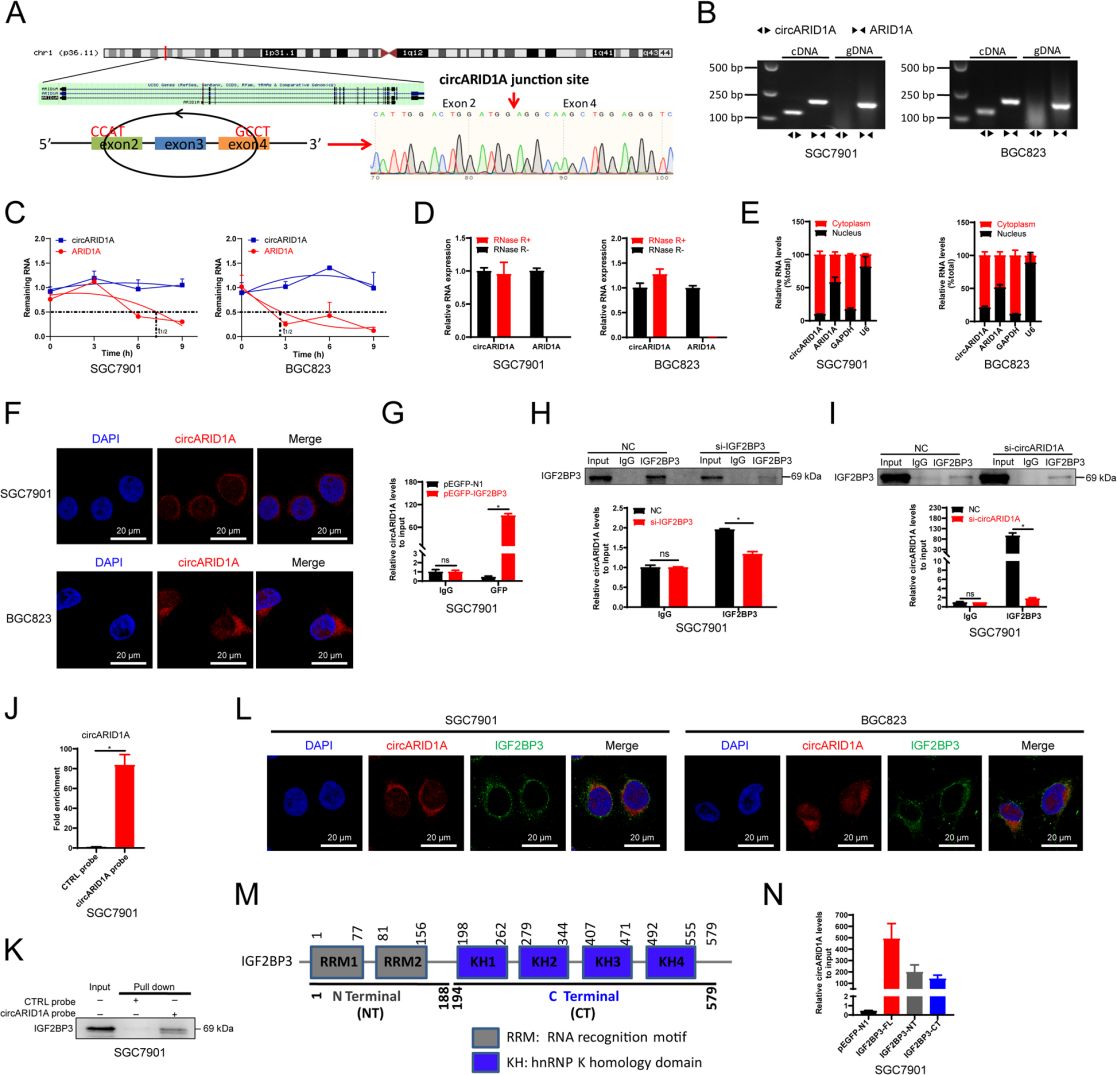
CircARID1A interacts with IGF2BP3 protein in GC cells. **A** Genomic locus of circARID1A in ARID1A. Sanger sequence of PCR products of circARID1A amplified in SGC7901 cells was shown. **B** Existence of circARID1A in cDNA and genomic DNA (gDNA) in GC cells. **C** Stability of circARID1A and ARID1A in SGC7901 and BGC823 cells treated with actinomycin D. **D** Expression of circARID1A and ARID1A for total RNA treated with RNase R. **E** Expression of circARID1A, ARID1A, GAPDH and snoU6 in nuclear and cytoplasmic fractions of SGC7901 and BGC823 cells. **F** CircARID1A location in SGC7901 and BGC823 cells evaluated by RNA-FISH. **G** RIP analysis of circARID1A enrichment pull-downs by GFP in SGC7901 cells overexpressing EGFP-tagged IGF2BP3. **H**-**I** RIP analyses of circARID1A enrichment pull-downs by IGF2BP3 in SGC7901 cells following (**H**) IGF2BP3 and (**I**) circARID1A knockdowns. **J** Enrichment efficiency of biotin tagged circARID1A probes assessed by RNA pull-downs. **K** Western blot validation of interaction of circARID1A and IGF2BP3 by RNA pull-down in SGC7901 cells. **L** RNA-FISH combined with IF demonstrating colocalization of circARID1A and IGF2BP3 in SGC7901 and BGC823 cells. **M** Schematic of IGF2BP3 binding domains. **N** RIP analysis of circARID1A enrichment pull-downs by GFP in SGC7901 cells transfected with full-length of IGF2BP3 or truncations. The *P*-values were calculated using the two-tailed Student’s t test. *P < 0.05

CircARID1A was derived from exons 2, 3 and 4 of ARID1A (Fig. 2A). To verify that circARID1A was circular RNA rather than products of trans-splicing or genomic rearrangements, circRNA identification assays were performed. Genomic DNA and RNA were extracted from SGC7901 and BGC823 cells and cDNA templates were synthesized using qRT-PCR amplification. Firstly, circARID1A was found only amplified from cDNA by divergent primers while no specific amplification product was observed from genomic DNA. In contrast, the linear RNA of ARID1A was amplified from both cDNA and genomic DNA by convergent primers (Fig. 2B). Moreover, we could identify the back-splice junction in PCR products of circARID1A from SGC7901 cells (Fig. 2A) and GC tissues or adjacent tissues (Supporting Information Fig. S5A-B). Actinomycin D assays indicated that the half-life of circARID1A was longer than that of ARID1A mRNA (Fig. 2C) and circARID1A was more RNase R resistant (Fig. 2D). As we know, the biological functions of circRNAs are dependent on cellular localization. We therefore sub-fractionated cells and circARID1A was primarily localized in the cytoplasm of SGC7901 and BGC823 cells (Fig. 2E-F).

For further validation of circARID1A-IGF2BP3 interaction, EGFP tagged IGF2BP3 was constructed and the RIP assays results showed that circARID1A could also bind to IGF2BP3 fusion expressed with EGFP in SGC7901 cells (the same sample as our published data in reference 16, Fig. 2G) or BGC823 cells (Supporting Information Fig. S6A). Moreover, the amount of circARID1A binding to IGF2BP3 decreased after IGF2BP3 or circARID1A knockdown in SGC7901 cells (Fig. 2H-I) or BGC823 cells (Supporting Information Fig. S6B-C). Subsequent circARID1A probes, but not the control probes, could enrich IGF2BP3 protein using RNA pull-down assays in SGC7901 cells (Fig. 2J-K), as well as in BGC823 cells (Supporting Information Fig. S6D-E). Additionally, RNA-FISH combined with IF assays demonstrated that circARID1A and IGF2BP3 proteins were colocalized in SGC7901 and BGC823 cells (Fig. 2L). IGF2BP3 is composed of 6 domains including 2 RRM motifs and 4 KH domains (Fig. 2M). We therefore generated pEGFP-N1 constructs with the marker in different locations in IGF2BP3 as well as truncations of the gene (Fig. 2M). RIP-PCR results (the same sample as our published data in reference 16) from truncations indicated that both the N- and C-termini of IGF2BP3 could interact with circARID1A in SGC7901 cells and especially the N-terminal truncation (Fig. 2N). However, circARID1A did not regulate IGF2BP3 expression in SGC7901 and BGC823 cells (Supporting Information Fig. S7A-B), as well as IGF2BP3 did not regulate the expression of circARID1A in BGC823 and AGS cells (Supporting Information Fig. S7C). Taken together, these results demonstrated that circARID1A is primarily cytoplasmic and interacts with IGF2BP3 protein.

### CircARID1A is upregulated in GC tissues and correlates with clinicopathological characteristics

**Fig. 3:**
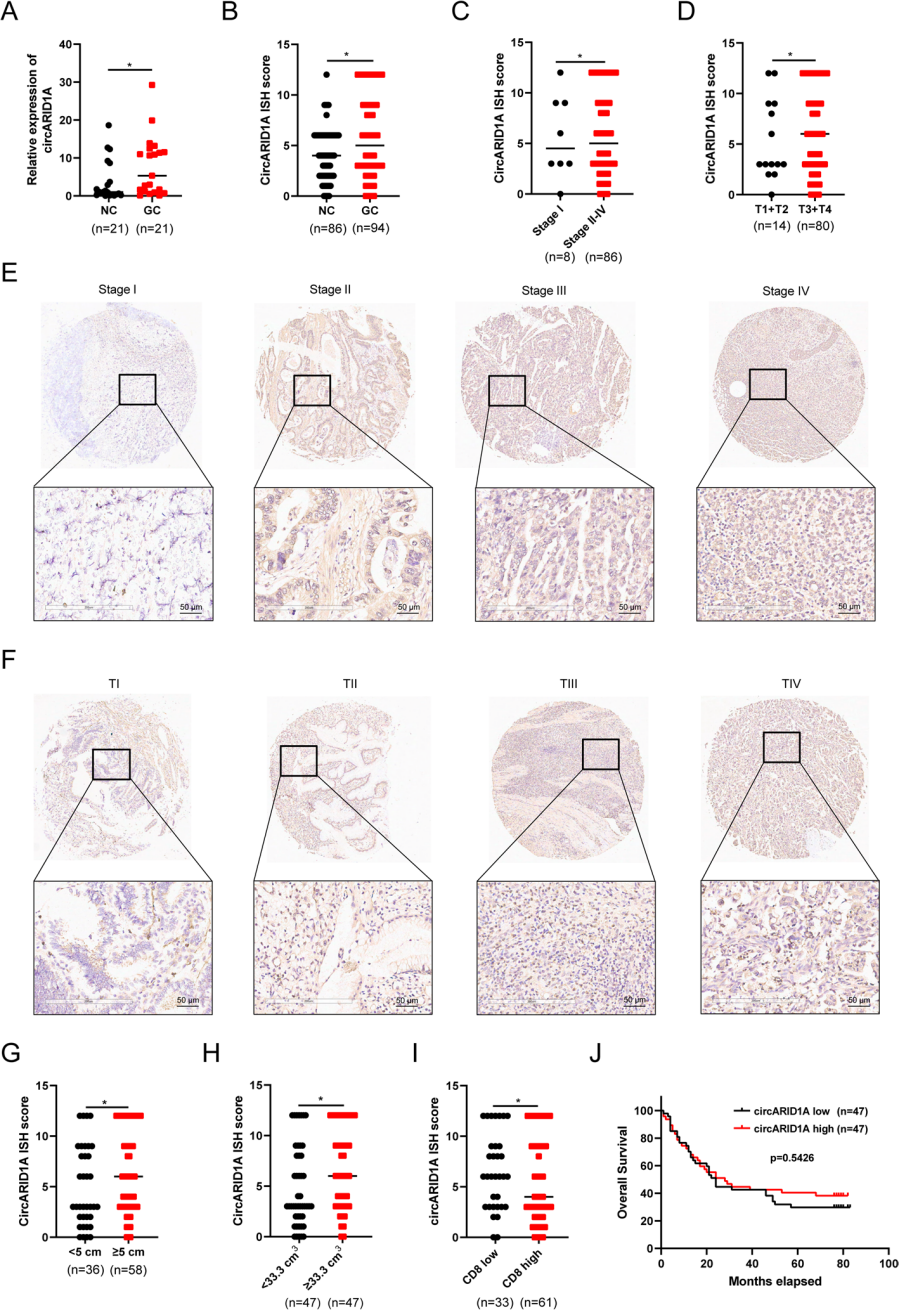
Expression of circARID1A in GC and associated clinical characteristics. **A** CircARID1A levels in GC tissues and paired adjacent tissues (*n* = 21). **B** ISH analysis of circARID1A expression in TMA of GC patients (adjacent tissues = 86 cases; GC tissues = 94 cases). **C** ISH analysis of circARID1A expression in GC patients with stage I and stage II-IV. **D** ISH analysis of circARID1A expression in GC patients with stage TI + TII and stage TIII + TIV. **E**–**F** Representative images of circARID1A staining in GC tissues at different TNM and T stages. **G** Expression of circARID1A in GC patients with tumor lengths < 5 cm or ≥ 5 cm. **H** Expression of circARID1A in GC patients with tumor volumes < 33.3 cm^3^ or ≥ 33.3 cm^3^. **I** ISH analysis of circARID1A expression in GC patients with low or high CD8 expression. **J** Overall survival analysis of GC patients with low or high expression of circARID1A. The *P*-values were calculated using the Wilcoxon Signed Rank Test. *P < 0.05

To uncover the expression of circARID1A in GC, a cohort containing 21 paired GC tissues was enrolled in the present study and we found that circARID1A was upregulated in GC tissues (Fig. 3A). To further clarify the association of circARID1A with clinical characteristics of GC, tissue microarrays containing 94 GC tissues and 86 peritumor tissues was employed to measure the expression of circARID1A in GC tissues by ISH (Supporting Information Table S10). The results indicated that the circARID1A ISH score was higher in GC tissues compared with peritumor tissues (Fig. 3B). Moreover, circARID1A expression was higher in GC tissues with advanced stage (stage II-IV Vs stage I) or higher T stage (T3 + T4 Vs T1 + T2) of disease (Fig. 3C-F). Furthermore, the GC patients were divided into two groups according to tumor length and tumor volume and we found that circARID1A expression was higher in patients with tumor lengths ≥ 5 cm or tumor volume ≥ 33.3 cm^3^ (Fig. 3G-H). Interestingly, circARID1A expression was higher in those GC tissues that displayed lower levels of CD8 expression (Fig. 3I). However, circARID1A expression was not associated with the overall survival of GC patients (Fig. 3J). In brief, circARID1A was significantly overexpressed in GC and associated with tumor TNM stage, T stage, tumor length and volume, indicating it might play an oncogenic role in GC.

### CircARID1A knockdown inhibits the proliferation of GC cells *in vitro* and *in vivo*

**Fig. 4:**
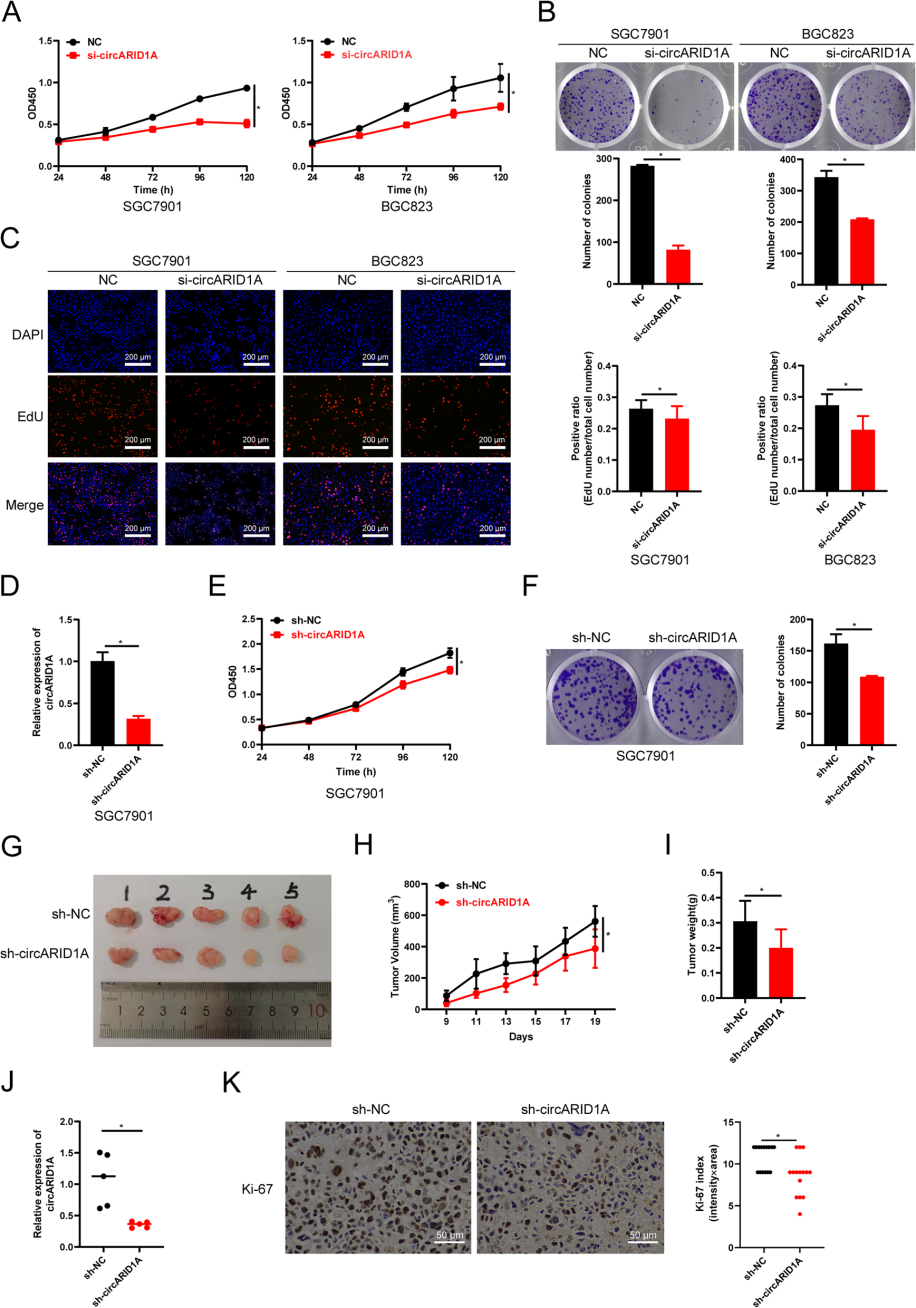
Knockdown of circARID1A inhibits the proliferation of GC *in vitro* and *in vivo*. **A** Cell viability of SGC7901 and BGC823 cells following circARID1A knockdown. **B**-**C** (**B**) Plate colony formation and (**C**) EdU assays of SGC7901 and BGC823 cells following circARID1A knockdown. **D** Efficiency of lentivirus sh-circARID1A transduction in SGC7901 cells. **E** Cell viability of SGC7901 cell following circARID1A stable knockdown. **F** Plate colony formation assay of SGC7901 cells with circARID1A stable knockdown. **G** Images of xenograft GC tumors with circARID1A knockdown. **H** Tumor volumes of xenograft GC tumors at different times. **I** Tumor weights of xenografts evaluated after mouse sacrifice. **J** CircARID1A expression in xenografted tumor tissues. **K** Representative images of Ki-67 expression evaluated by IHC in xenografted tumor tissues. Three different visual fields were randomly selected for each slice. The *P*-values were calculated using the two-tailed Student’s t test. *P < 0.05

CircARID1A is a novel circRNA in GC and we then investigated its biological roles in GC. We first constructed a small interfering RNA (siRNA) targeting the back-splicing region of circARID1A. The results showed that we successfully manipulated the expression of circARID1A in SGC7901 and BGC823 cells without affecting the mRNA levels of ARID1A (Supporting Information Fig. S8A-B). The viability of SGC7901 and BGC823 cells was significantly inhibited after circARID1A knockdown (Fig. 4A). The plate colony formation and EdU assays indicated SGC7901 and BGC823 proliferation were also suppressed after circARID1A knockdown (Fig. 4B-C). These results revealed that circARID1A enhanced the proliferation of GC *in vitro*. In order to explore the role of circARID1A overexpression in GC, we generated a vector containing the entire circARID1A sequence in the vectors pLC5-ciR, pLV-ciR and pcDNA3.1( +) CircRNA Mini Vector. However, no significant overexpression of circARID1A could be found in SGC7901 and BGC823 cells transfected with these overexpressing plasmids (Supporting Information Fig. S9A-C). In order to rule out whether the circARID1A sequence affects the cyclization reaction in cells we used an unrelated circRNA, circPDHK1, as a control. We found significant increased expression of circPDHK1 in SGC7901 and BGC823 cells transfected with pLC5-ciR-circPDHK1 vector (Supporting Information Fig. S9D). These results suggested that exogenous circARID1A engineered through overexpression constructs was inhibited in GC cell lines for unknown reasons.

Subsequently, SGC7901 cells containing a stable circARID1A knockdown were constructed by lentivirus infection (Fig. 4D). Consistent with the function of circARID1A interference by siRNA, proliferation of these cells was inhibited (Fig. 4E-F). To further evaluate the oncogenic role of circARID1A *in vivo*, BABL/c nude mice (3 weeks old) were employed to establish a model of implanted tumor model of human GC. CircARID1A knockdown resulted in decreased sizes of subcutaneously implanted tumors (Fig. 4G) as well as the tumor volume and tumor weight (Fig. 4H-I). Additionally, circARID1A and Ki-67 were both down-regulated in these tumors with circARID1A knockdown (Fig. 4J-K). Taken together, these results revealed that circARID1A promoted the proliferation of GC *in vitro* and *in vivo*.

### CircARID1A is a key factor for the proliferation promotion effect of IGF2BP3 in GC

**Fig. 5:**
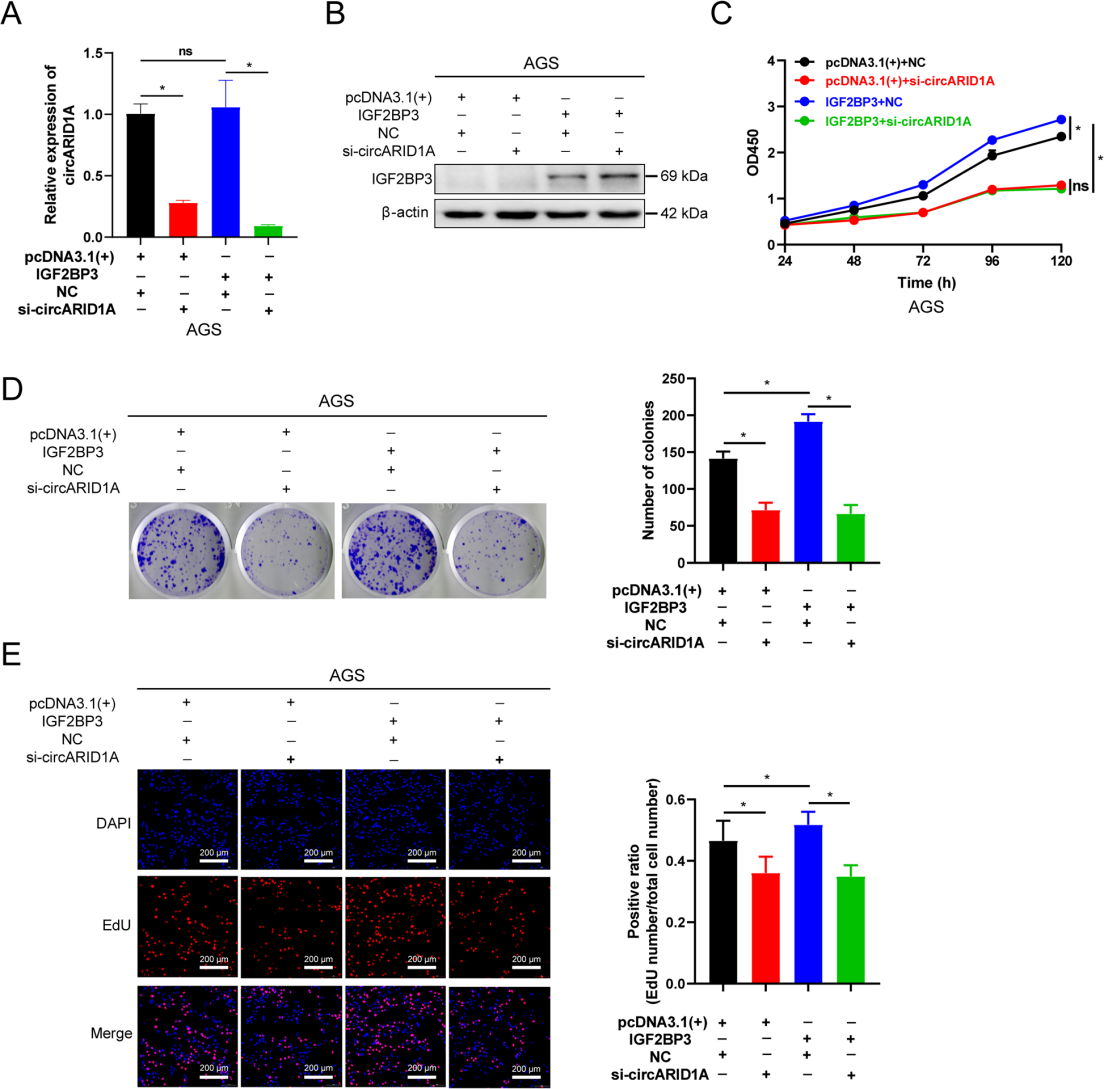
CircARID1A interacts with IGF2BP3 protein to promote the proliferation of GC *in vitro*. **A-B** (**A**) Interference efficiency of si-circARID1A and (**B**) overexpression efficiency of IGF2BP3 in AGS cells. **C**-**E** (**C**) CCK-8, (**D**) plate colony formation, and (**E**) EdU assays of AGS cells transfected with vector or IGF2BP3 and co-transfected with NC or si-circARID1A as indicated. The *P*-values were calculated using one way ANOVA. *P < 0.05

The above results demonstrated that circARID1A can directly bind IGF2BP3 but whether this binding regulates GC proliferation was unclear. Here, we selected AGS cells for functional rescue experiment because the expression of IGF2BP3 protein in AGS cells was relative lower than in other GC cell lines (Supporting Information Fig. S1C). We therefore measured proliferation of AGS cells following circARID1A knockdowns and/or IGF2BP3 overexpression (Fig. 5A-B). AGS cell viability was increased after IGF2BP3 overexpression and inhibited after circARID1A knockdown. Surprisingly, the proliferation promotion effect of IGF2BP3 in AGS cells was decreased when circARID1A was knocked-down (Fig. 5C). The role of circARID1A or IGF2BP3 alone or combination in AGS cells evaluated by EdU assay and plate colony formation assay were consistent with the results from CCK-8 assay (Fig. 5D-E). These results demonstrated that the binding between circARID1A and IGF2BP3 is a key factor of the oncogenic role of IGF2BP3 in GC.

### CircARID1A interacts with IGF2BP3 to regulate the expression of SLC7A5 in GC

**Fig. 6:**
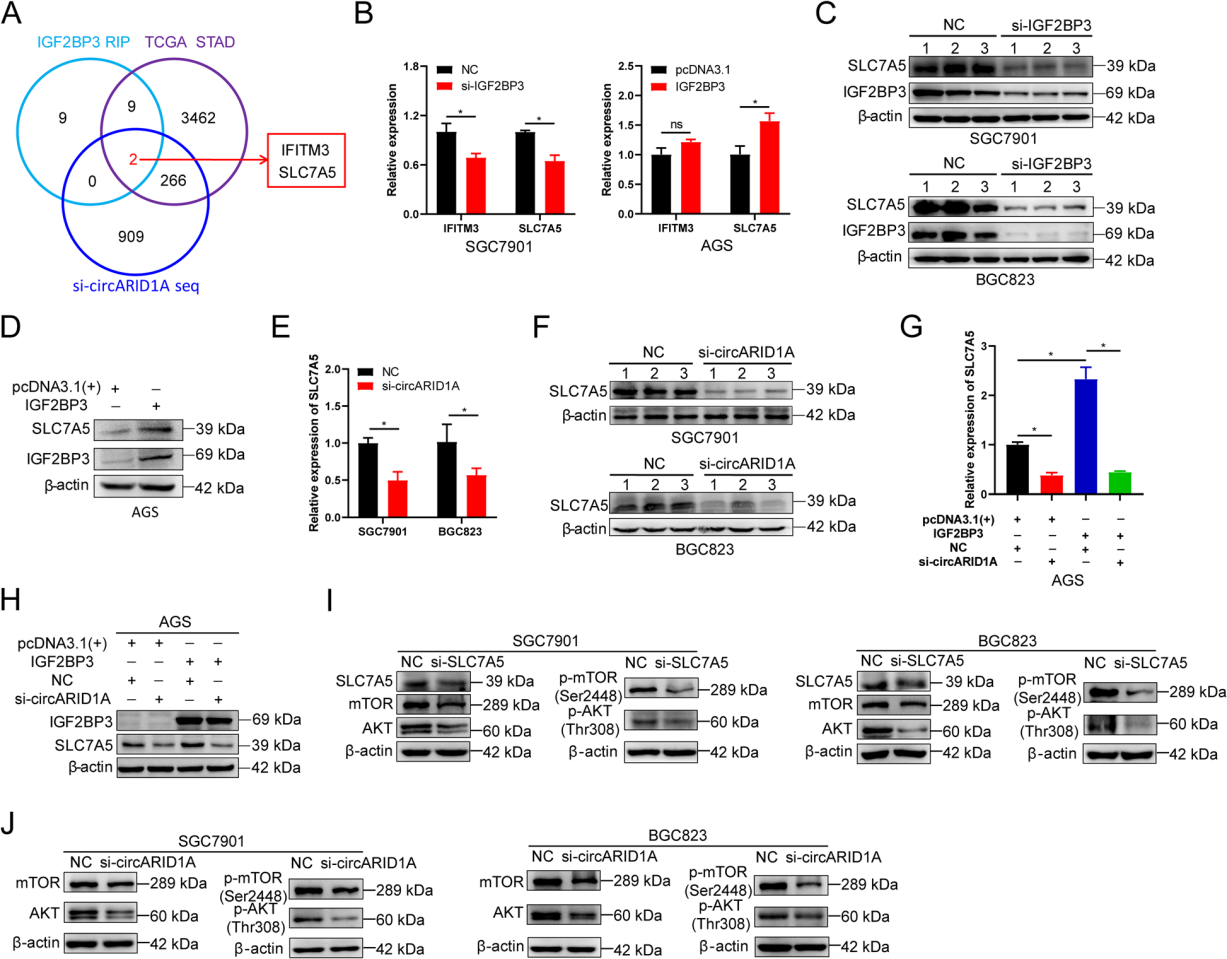
CircARID1A and IGF2BP3 regulate the expression of SLC7A5 in GC cells. **A** Potential circARID1A-IGF2BP3 targets. **B** Expression of IFITM3 and SLC7A5 mRNA in SGC7901 following IGF2BP3 knockdown and in AGS cells following IGF2BP3 overexpression. **C** Expression of SLC7A5 protein in SGC7901 and BGC823 cells following IGF2BP3 knockdown. **D** Expression of SLC7A5 protein in AGS cells following IGF2BP3 overexpression. **E**–**F** Expression of SLC7A5 (**E**) mRNA and (**F**) protein in SGC7901 and BGC823 cells following circARID1A knockdown. **G**-**H** Expression of SLC7A5 (**G**) mRNA and (**H**) protein in AGS cells transfected with vector or IGF2BP3 and co-transfected with NC or circARID1A. **I**-**J** Expression of AKT, mTOR, p-AKT and p-mTOR in SGC7901 and BGC823 cells following (**I**) SLC7A5 knockdown and (**J**) following circARID1A knockdown. The *P*-values were calculated using the two-tailed Student’s t test or one way ANOVA. *P < 0.05

IGF2BP3 regulates mRNA expression in normal and cancer cells so we attempted to identify the target mRNAs regulated by circARID1A-IGF2BP3. We firstly identified target genes regulated both by IGF2BP3 and circARID1A from the overlapping genes of three datasets that included the upregulated genes in STAD of the TCGA database, the top 20 mRNAs that binding to IGF2BP3 in SGC7901 cells from RIP-seq data and downregulated mRNAs in SGC7901 cells with circARID1A knockdown (Fig. 6A, Supporting Information Tables S8, S11 and S12). IFITM3 and SLC7A5 were identified and subsequently validated by RT-PCR regulated by circARID1A-IGF2BP3. IFITM3 and SLC7A5 mRNA levels were decreased in SGC7901 cells following IGF2BP3 knockdown while only SLC7A5 mRNA was elevated after IGF2BP3-forced expression in AGS cells (Fig. 6B). In addition, SLC7A5 protein levels were significantly decreased following IGF2BP3 knockdown in SGC7901 and BGC823 cells (Fig. 6C) and was increased in AGS, SGC7901, and BGC823 cells overexpressing IGF2BP3 (Fig. 6D, Supporting Information Fig. S10). Moreover, SLC7A5 mRNA and protein were largely inhibited in SGC7901 and BGC823 cells that contained the circARID1A knockdown (Fig. 6E-F).

AGS cells were then employed to evaluate the interaction between circARID1A and IGF2BP3 in regulating the expression of SLC7A5. We found that SLC7A5 mRNA and protein levels were significantly downregulated or upregulated when circARID1A was knocked-down or IGF2BP3 was overexpressed, respectively. The upregulation of SLC7A5 by IGF2BP3 was diminished when circARID1A was knocked-down (Fig. 6G-H). These data suggested that circARID1A plays a vital role in IGF2BP3 regulates the expression of SLC7A5.

Previous studies have demonstrated that SLC7A5 regulates AKT/mTOR pathway expression in breast cancer [30]. We found that knockdown of SLC7A5 in SGC7901 and BGC823 cells inhibited the expression of AKT, p-AKT, mTOR, and p-mTOR (Fig. 6I). Given that circARID1A regulated the expression of SLC7A5 in GC, we also measured the expression of these key proteins in GC cells after circARID1A knockdown. The results indicated that the expression of AKT, p-AKT, mTOR, and p-mTOR were also significantly inhibited after circARID1A was knocked-down in SGC7901 and BGC823 cells (Fig. 6J). Taken together, these results suggested that circARID1A-IGF2BP3 regulate the expression of SLC7A5 and AKT/mTOR pathway in GC.

### CircARID1A regulates the stability of SLC7A5 by forming circARID1A-IGF2BP3-SLC7A5 RNA–protein ternary complex

**Fig. 7:**
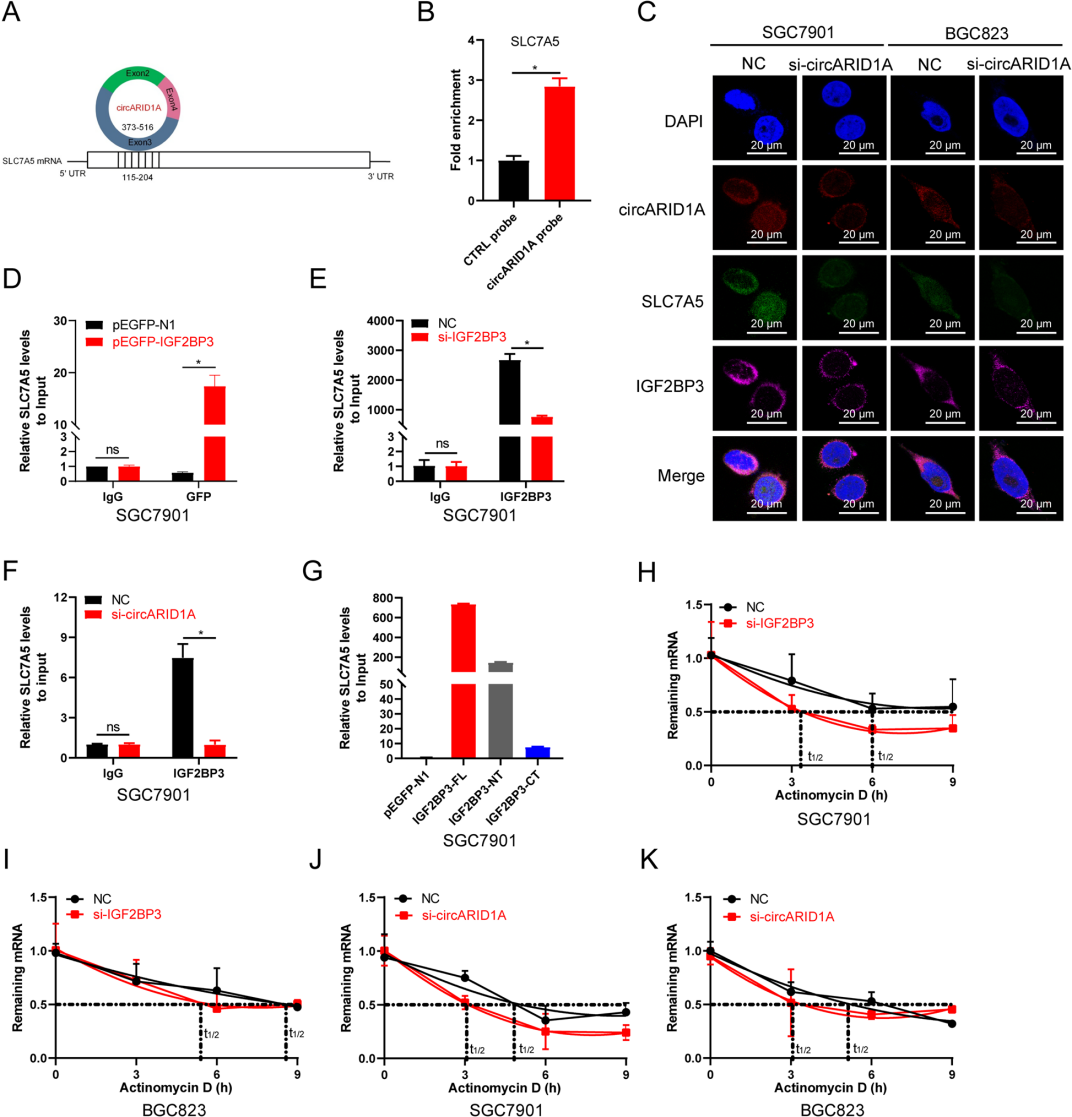
CircARID1A-IGF2BP3-SLC7A5 RNA–protein ternary complex regulates the stability of SLC7A5 mRNA in GC. **A** CircARID1A and SLC7A5 mRNA binding sites predicted using IntaRNA 2.0. **B** Enrichment of SLC7A5 mRNA by RNA pull-down using circARID1A probes. **C** RNA-FISH combined with IF to visualize the location of circARID1A, SLC7A5 and IGF2BP3 in SGC7901 and BGC823 cells with or without circARID1A knockdown as indicated. **D** RIP analysis of SLC7A5 enrichment using GFP pull-downs in SGC7901 cells overexpressing EGFP-tagged IGF2BP3 in SGC7901 cells. **E**–**F** RIP analysis of SLC7A5 enrichment using IGF2BP3 pull-downs in SGC7901 cells after (**E**) IGF2BP3 and (**F**) circARID1A knockdowns as indicated. **G** RIP analysis of SLC7A5 enrichment using GFP pull-downs in SGC7901 cells transfected with full-length of IGF2BP3 or truncations as indicated. **H**–**K** Stability of SLC7A5 mRNA in SGC7901 and BGC823 cells following (**H**-**I**) IGF2BP3 knockdown and actinomycin D treatment and (**J**-**K**) following circARID1A knockdown and actinomycin D treatment. The *P*-values were calculated using the two-tailed Student’s t test. *P < 0.05

The above results demonstrated that both circARID1A and IGF2BP3 can regulate SLC7A5 expression while the exact mechanism remains to be clarified. Non-coding RNAs have been shown to target mRNAs via direct or indirect RNA-RNA interactions [42, 43]. We therefore predicted the binding site between circARID1A and SLC7A5 mRNA using the IntaRNA 2.0 web tool (http://rna.informatik.uni-freiburg.de/IntaRNA) and found that the nucleotides at positions + 373–516 in circARID1A could bind to SLC7A5 mRNA at + 115–204 and the predicated binding strength was high at -76.54 kcal/mol (Fig. 7A). RNA pull-down assay also indicated that circARID1A probes could enrich for SLC7A5 mRNA over that of control probes (Fig. 7B). Moreover, RNA-FISH combined with IF assays indicated that circARID1A, SLC7A5 and IGF2BP3 colocalized in SGC7901 and BGC823 cells, and the colocalization was reduced after circARID1A knockdown (Fig. 7C). These results revealed that circARID1A, IGF2BP3 and SLC7A5 might formed an RNA–protein ternary complex.

Next, RIP assay was employed to further validate the circARID1A-IGF2BP3-SLC7A5 RNA–protein ternary complex in GC cells. We firstly evaluated whether exogenous IGF2BP3 could bind to SLC7A5 mRNA using a EGFP tagged IGF2BP3. The results demonstrated that EGFP-IGF2BP3 fusion proteins captured more SLC7A5 mRNA than EGFP expressing vector only (Fig. 7D). Moreover, SLC7A5 binding to IGF2BP3 was reduced after IGF2BP3 or circARID1 knockdown in SGC7901 cells (Fig. 7E-F). Given that SLC7A5 mRNA could bind to IGF2BP3, we then used IGF2BP3 truncations to verify the binding site of SLC7A5 mRNA in IGF2BP3. Our results indicated that SLC7A5 mRNA dominantly interacted with the N-terminal of IGF2BP3 (Fig. 7G). These results demonstrated that circARID1A, SLC7A5 and IGF2BP3 formed an RNA–protein ternary complex in GC cells.

Since IGF2BP3 can regulate gene expression by affecting mRNA stability [44], we measured the stability of SLC7A5 mRNA in actinomycin D-treated SGC7901 and BGC823 cells after IGF2BP3 or circARID1A knockdowns. The half-life of SLC7A5 mRNA was shorter in SGC7901 and BGC823 cells treated with IGF2BP3 interference (Fig. 7H-I) as well as circARID1A knockdown (Fig. 7J-K). Overall, these results demonstrated that circARID1A-IGF2BP3-SLC7A5 RNA–protein ternary complex regulated the stability of SLC7A5 mRNA in GC cells.

### CircARID1A promotes GC proliferation in a SLC7A5-dependent manner

**Fig. 8:**
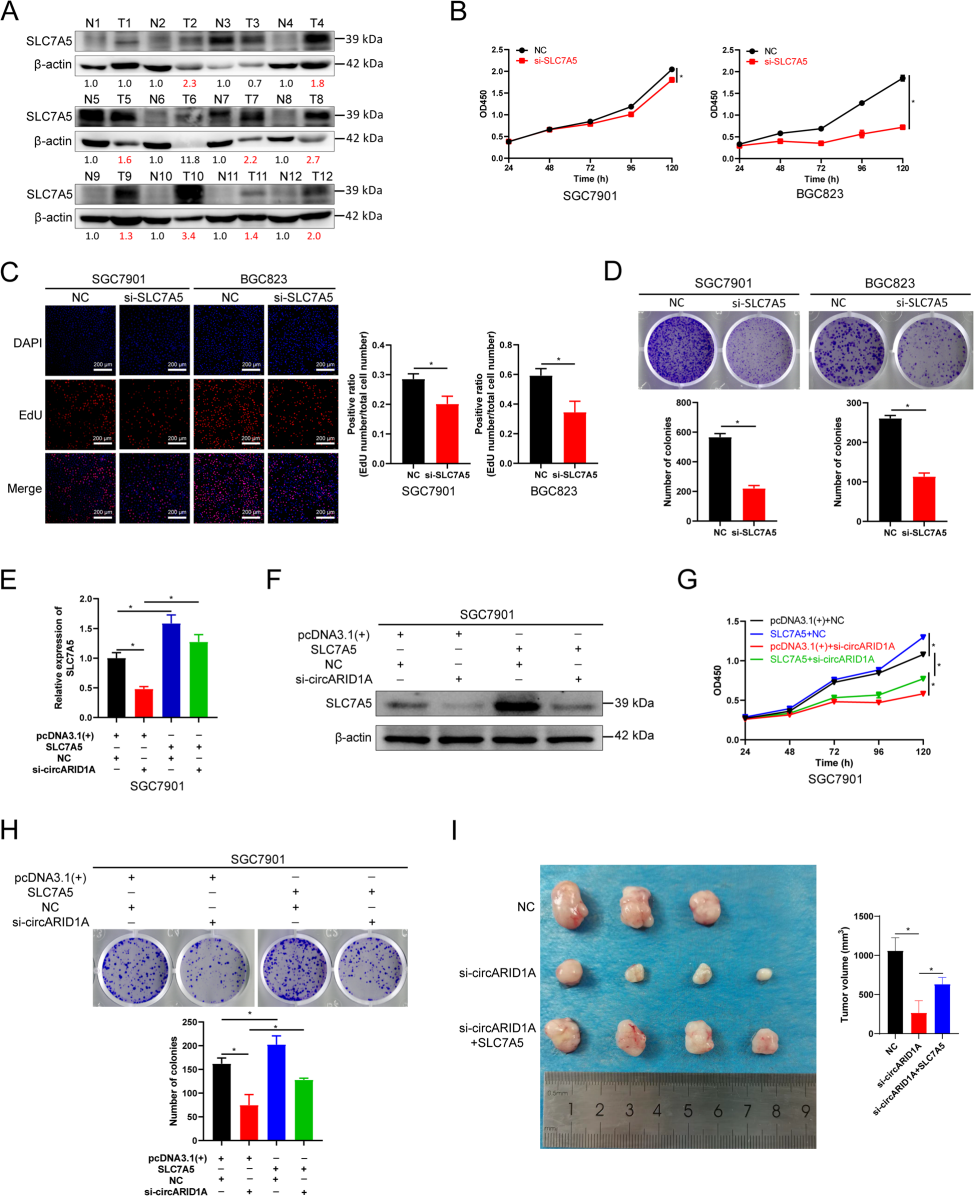
CircARID1A promotes the proliferation of GC by upregulating SLC7A5. **A** Expression of SLC7A5 in 12 paired GC tissues. **B** CCK-8 assay of SGC7901 and BGC823 cells following SLC7A5 knockdown. **C**-**D** (**C**) EdU and (**D**) plate colony formation assays in SGC7901 and BGC823 cells following SLC7A5 knockdown. **E**–**F** The expression of SLC7A5 (**E**) mRNA and (**F**) protein in SGC7901 cells transfected with vector or SLC7A5 and those co-transfected with NC or si-circARID1A. **G**-**H** (**G**) CCK-8 or (**H**) plate colony formation assay of SGC7901 cells transfected with vector or SLC7A5 and those co-transfected with NC or si-circARID1A as indicated. **I** *In vivo* analyses of tumor in mice that were subcutaneously implanted with HGC-27 cells and injected with Negative control, cholesterol-modified si-circARID1A combined with or without lentivirus containing SLC7A5. The *P*-values were calculated by two-tailed Student’s t test or one way ANOVA. *P < 0.05

Next, we evaluated the expression and function of SLC7A5 in GC. SLC7A5 mRNA levels are significantly upregulated in GC tissues of TCGA database (Supporting Information Fig. S11A). In our cohort, SLC7A5 expression was also upregulated in most of the GC tissues (9/12) (Fig. 8A). In GC cell lines, the expression of SLC7A5 was also upregulated in SGC7901, BGC823, AGS and MKN74 cells compared with normal gastric epithelial cell line GES-1 (Supporting Information Fig. S11B). Moreover, expression of SLC7A5 was positively correlated with IGF2BP3 in GC tissues from TCGA database (Supporting Information Fig. S11C). These results revealed that SLC7A5 levels are elevated in GC.

Next, the role of SLC7A5 in GC was evaluated. The viability of SGC7901 and BGC823 cells was inhibited after SLC7A5 knockdowns (Fig. 8B). Moreover, proliferation of SGC7901 and BGC823 cells was also suppressed after SLC7A5 inhibition (Fig. 8C-D). Furthermore, overexpression of SLC7A5 significantly counteracted the si-circARID1A-induced SLC7A5 downregulation (Fig. 8E-F). As expected, SLC7A5 overexpression rescued the decreased proliferation of SGC7901 cells caused by circARID1A knockdown (Fig. 8G-H). Additionally, HGC-27 cells were employed for in vivo rescue assay of circARID1A and SLC7A5. Firstly, we confirmed the inhibition effect of circARID1A knockdown on the viability and proliferation of HGC-27 cells (Supporting Information Fig. S12A-C). Consistently, lentivirus-mediated overexpression of SLC7A5 obviously rescued the inhibitory effect of cholesterol-modified si-circARID1A on the growth of HGC-27 cells-derived tumor xenograft (Fig. 8I). Taken together, the regulatory role of circARID1A on GC proliferation was SLC7A5-dependent.

## Discussion

Previous studies have demonstrated that IGF2BP3 is dysregulated in a variety of tumors [45]. In gastric cancer, IGF2BP3 is upregulated and positively associated with lymphoid metastasis, high Ki-67 expression and poor outcome [46]. Moreover, IGF2BP3 was also considered as an independent poor prognostic factor and a predictor of recurrence after surgery in GC [14]. Furthermore, our previous study revealed that IGF2BP3 could promote the metastasis of GC as well [16]. Mechanistically, IGF2BP3 could serve as an m6A “reader” to recognize m6A modified mRNA and enhance HDGF mRNA stability to promote tumor angiogenesis and glycolysis in GC [15]. Zhou et al. also revealed the oncogenic role of IGF2BP3 and confirmed IGF2BP3 was the target of miR-34a in gastric carcinogenesis [17]. In the present study, we consistently validated that IGF2BP3 expression was upregulated in GC tissues based on our results and the TCGA data, and was associated with a poor prognosis. Our further gain- and loss-of-function studies indicated that IGF2BP3 promoted proliferation of GC suggesting that IGF2BP3 plays an oncogenic role in GC. This urged us to investigate the molecular mechanisms of IGF2BP3 actions.

There is increasing evidence that circRNAs play vital roles in tumorigenesis and progression of GC. For example, circST3GAL6 inhibited the malignant behaviors of GC cells through the miR-300/FOXP2 axis and regulated apoptosis and autophagy [47]. CircRAB31 acted as a miR-885-5p sponge to suppress GC proliferation and metastasis via the PTEN/PI3K/AKT pathway [22]. Moreover, circRNAs could also interact with IGF2BP3 to regulate GC progression. For instance, our previous study demonstrated that circTNPO3 competitively interacted with IGF2BP3 to inhibit MYC and Snail expression and finally suppressed GC proliferation and metastasis [16]. Furthermore, IGF2BP3-circRNA interactions have been found in other types of cancer such as hsa_circ_0003258-IGF2BP3, CDR1as-IGF2BP3 and hsa_circ_0000231-IGF2BP3 [35, 48, 49]. Given that abundant noncoding RNAs exist in eukaryotes [50, 51], a comprehensive identification of RNAs interacting with IGF2BP3 is helpful to understand the oncogenic role of IGF2BP3 in GC. In the present study, RIP combined with RNA sequencing uncovered a series of RNAs, including mRNAs and circRNAs, that interacted with IGF2BP3 in GC. A novel circRNA derived from the pre-mRNA of ARID1A was identified in GC and termed circARID1A. Subsequently, qRT-PCR and tissue microarrays indicated that circARID1A was significantly increased in GC tissues compared with noncancerous tissues and was positively associated with tumor length, tumor volume, T stage and TNM stage. Additionally, circARID1A expression levels were higher in GC tissues with lower CD8 expression indicating that circARID1A might be involved in modulation of the tumor immune microenvironment. Next, functional results indicated that circARID1A promoted GC growth *in vitro* and *in vivo*. These results implicated circARID1A as a potential therapeutic target for GC.

Ideally, functional study of circARID1A requires both knockdown and overexpression of circARID1A. In the current work, we firstly constructed an overexpression vector for circARID1A based on the pLC5-ciR plasmid, a commercial vector specific for circRNA overexpression. No matter how we improved the conditions, no overexpression of circARID1A was observed in GC cell lines. A previous study demonstrated that the biogenesis of exon derived circRNA requires intronic repeats to collaborate with the exons, as well as the sequence or length of exon was also main factors affecting the circularization of exons [52]. Moreover, short intronic repeats was necessary for the circularization of some exons [52]. We next employed other commercial vectors including pCD5-ciR, pLV-ciR and pcDNA3.1( +)-circRNA Mini Vector to construct circARID1A overexpression vectors, while none of these vectors worked. In order to rule out whether the circARID1A sequence may affect the cyclization reaction in cells, we used an unrelated circRNA, circPDHK1, as the control. We constructed a vector by inserting circPDHK1 into the pLC5-ciR plasmid (pLC5-ciR-circPDHK1) and found a remarkable elevation of circPDHK1 after GC transfection. These results revealed that the base composition of circARID1A might be one of the factors that inhibits the circularization of circARID1A expressed by these vectors in different cell types.

CircRNA-protein interactions are one way that circRNAs regulate tumor biological behaviors. NcRNA functions are tightly and closely associated with subcellular location [53–55]. CircECE1 was predominantly localized within the cytoplasm and interacted with c-Myc to suppress the degradation of c-Myc through the proteasome, subsequently activated the Warburg effect and promoted osteosarcoma progression [56]. In addition to dominant localization in the cytoplasm, some circRNAs are located in the nucleus. For example, circLRIG3 preferentially located in the nucleus and interacts with both EZH2 and STAT3 in hepatocellular carcinoma to form a circRNA-protein complex, facilitating EZH2-induced activation of STAT3 signaling and promoted HCC cell proliferation and metastasis [57]. CircURI1 was reported to preferentially locate in the nucleus in both AGS and SGC7901 cells. Mechanistically, circURI1 interacted with hnRNPM to regulate the alternative splicing of VEGFA and subsequently inhibited the metastasis of GC [58]. In the present study, we found that circARID1A was dominantly located in the cytoplasm of GC and interacted with IGF2BP3, which was also located in cytoplasm. Moreover, circARID1A could interact with both the N- and C-terminal regions of IGF2BP3, especially the N-terminus. However, which region of circARID1A could bind to IGF2BP3 needs to be further investigated when the problem of circARID1A overexpression is solved. Functionally, the oncogenic role of IGF2BP3 in GC required the participation of circARID1A and knockdown of circARID1A abolished the proliferation promotion effect of GC induced by IGF2BP3 overexpression. These results revealed that the binding of circARID1A and IGF2BP3 was a key factor of the oncogenic role of IGF2BP3 in GC.

CircRNAs play crucial roles in the regulation of downstream gene expression via several mechanisms including miRNA sponge effects or regulating transcription [59, 60]. For instance, circCYFIP2 served as a ceRNA of miR-1205 to regulate the expression of E2F1 and promote GC progression [59]. In addition, circHuR interacted with CNBP and subsequently restrained its binding to the HuR promoter, resulting in downregulation of HuR and repression of tumor progression [60]. But what's remarkable is that most circRNAs bind to miRNAs or a protein alone. In the present study, we identified that SLC7A5 mRNA was the common target of circARID1A and IGF2BP3. Amazingly, we validated that circARID1A and IGF2BP3 could directly interact with SLC7A5 mRNA and form a circARID1A-IGF2BP3-SLC7A5 RNA–protein ternary complex; this enhanced SLC7A5 mRNA stability that elevated its expression in GC and finally promoted GC proliferation. Consistent with our study, circDCUN1D4 was also reported to act as a scaffold to facilitate the interaction between the HuR protein and TXNIP mRNA to form circDCUN1D4/HuR/TXNIP RNA–protein ternary complex. This complex enhanced the stability of the TXNIP mRNA and suppressed metastasis and glycolysis of lung adenocarcinoma [61].

Previous studies have shown that SLC7A5 is highly expressed in breast cancer, and positively associated with histopathological grade and the expression of proliferation marker Ki-67 and HIF-1α. Targeting SLC7A5 amino acid transport activity using JPH203 significantly suppressed the proliferation of breast cancer [31]. In Non-Hodgkin lymphoma, SLC7A5 expression was also significantly upregulated and was inversely correlated with patient survival span [28]. In our study, SLC7A5 was increased remarkably in GC and this contributed to GC proliferation. Moreover, overexpression of SLC7A5 rescued the role of circARID1A knockdown on the expression of SLC7A5 and GC proliferation. A previous study also showed that SLC7A5 regulated AKT/mTOR activation in breast cancer [30]. In our study, the AKT and mTOR, as well as p-AKT and p-mTOR, were inhibited after SLC7A5 knockdown in SGC7901 and BGC823 cells. This indicated that SLC7A5 was the downstream target of circARID1A in GC.

## Conclusions

**Fig. 9:**
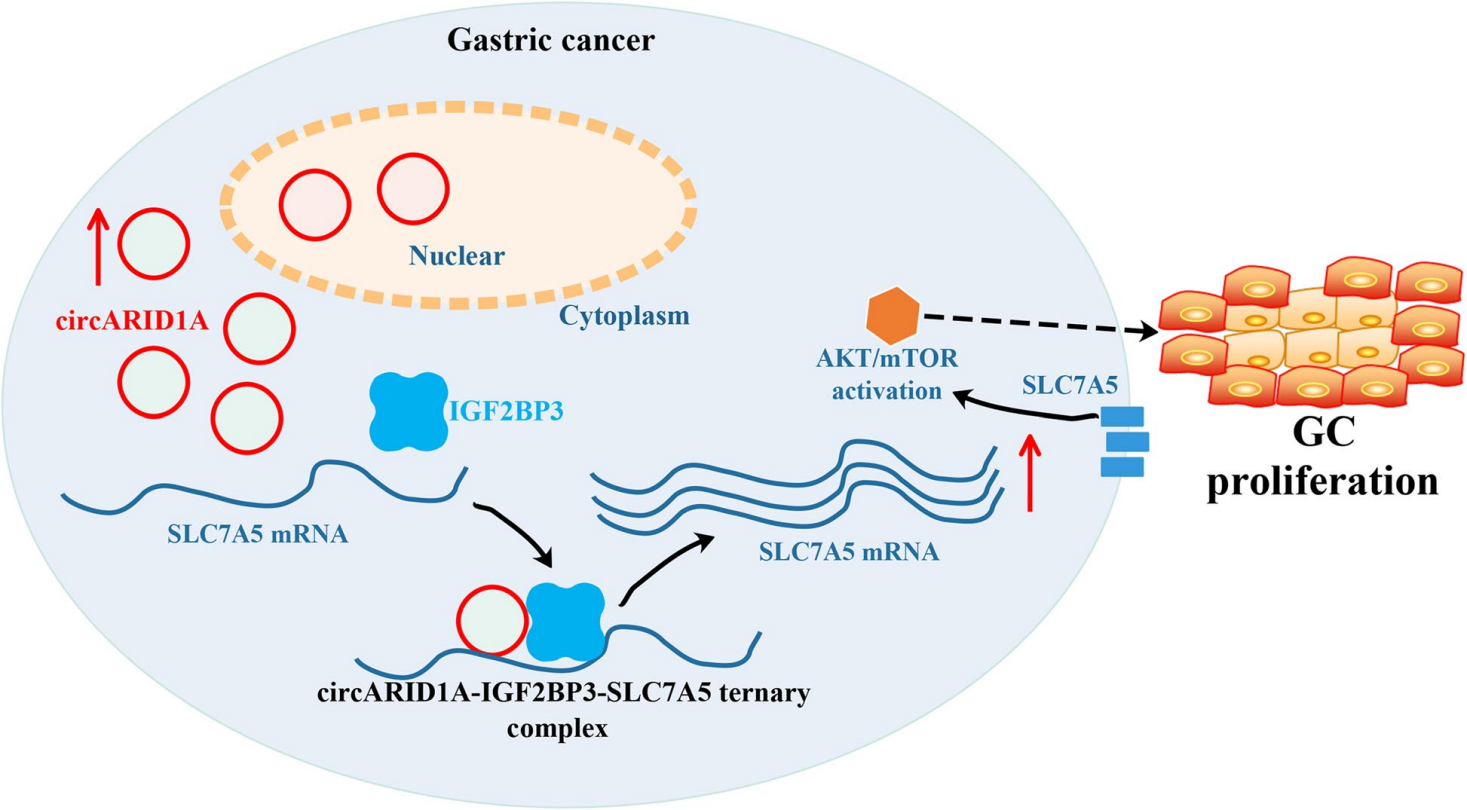
A schematic illustration of the molecular mechanism of circARID1A in promoting the proliferation of GC

In the present study, we identified a novel circRNA, circARID1A, which bound to IGF2BP3 in GC. CircARID1A was upregulated in GC tissues and the level of circARID1A was associated with tumor length, tumor volume, TNM stage, and T stage. CircARID1A promoted the proliferation of GC cells *in vitro* and *in vivo*. Mechanistically, circARID1A promoted the proliferation of GC by forming a circARID1A-IGF2BP3-SLC7A5 RNA–protein ternary complex, enhancing the stability of SLC7A5 mRNA and regulating AKT/mTOR pathway. Moreover, circARID1A was a key factor of the oncogenic role of IGF2BP3 in GC. Small molecule inhibitors or biotherapy targeting circARID1A-IGF2BP3-SLC7A5 axis might be a novel strategy for GC treatment (Fig. 9).

## Supplementary Information

**Additional file 1: Table S1.** Characteristics of GC patients for circARID1A measurement. **Table S2.** SiRNAs sequences. **Table S3.** Probes for RNA-FISH. **Table S4.** Primers for amplification of indicated genes. **Table S5.** Probes for RNA pull-down. **Table S6.** Probes targeting circARID1A for TMA. **Table S7.** RBPs list. **Table S8.** Differential expression genes in STAD of the TCGA database. **Table S9.** CircRNAs interacting with IGF2BP3 in SGC7901 cells (Top 20). **Table S10.** Characteristics of GC patients for TMA. **Table S11.** Downregulated genes evaluated by RNA-seq in SGC7901 cells following circARID1A knockdown. **Table S12.** RNAs interacting with IGF2BP3 in SGC7901 cells evaluated by RIP-seq (Top 20).

**Additional file 2**: **Fig. S1** IGF2BP3 expression in GC tissues or cell lines. A Heat map of RBPs dysregulated in STAD of TCGA database. B Representative IHC images of IGF2BP3 expression in normal gastric mucosa or GC tissues from HPA database. C The expression of IGF2BP3 in normal gastric epithelia cell GES-1 and gastric cancer cell lines SGC7901, BGC823, AGS, and MKN74. **Fig. S2** The interference efficiency and inhibiting proliferation effect of two designed IGF2BP3 siRNAs. A The interference efficiency of si-IGF2BP3-1 and si-IGF2BP3-2 in SGC7901 and BGC823 cells. B The viability of SGC7901 and BGC823 cells transfected with si-IGF2BP3-1 and si-IGF2BP3-2. C The proliferation of SGC7901 and BGC823 cells transfected with si-IGF2BP3-1 and si-IGF2BP3-2. The *P*-values were calculated using the two-tailed Student’s t test. *P < 0.05. **Fig. S3** The expression of IGF2BP3 in SGC7901 and BGC823 cells after IGF2BP3 knockdown or overexpression. A The expression of IGF2BP3 in SGC7901 and BGC823 cells after IGF2BP3 knockdown by siRNA. B The expression of IGF2BP3 in SGC7901 and BGC823 cells with IGF2BP3 stable overexpression. **Fig.** **S4** Screening of circRNAs that binding to IGF2BP3 protein in GC. A Heat map of circRNAs binding to IGF2BP3 in SGC7901 cells evaluated by RIP-seq. B The expression of circELK4 in GC tissues (GC) and adjacent tissues (NC). The *P*-value was calculated using the two-tailed Student’s t test. **Fig. S5** CircARID1A sequencing results of GC tissues and the adjacent tissues. A CircARID1A sequencing results of GC tissue and the adjacent tissue from patient 1. B CircARID1A sequencing results of GC tissue and the adjacent tissue from patient 2. **Fig. S6** Validation of the binding between circARID1A and IGF2BP3 proteins in BGC823 cells. A RIP analysis of circARID1A enrichment pull-downs by GFP in BGC823 cells overexpressing EGFP-tagged IGF2BP3. B-C RIP analyses of circARID1A enrichment pull-downs by IGF2BP3 in BGC823 cells following (B) IGF2BP3 or (C) circARID1A knockdowns. D Enrichment efficiency of biotin tagged circARID1A probes assessed by RNA pull-downs. E Western blot validation of interaction of circARID1A and IGF2BP3 by RNA pull-down in BGC823 cells. The *P*-values were calculated using the two-tailed Student’s t test. *P < 0.05. **Fig. S7** Regulation of circARID1A and IGF2BP3 to each other in GC cells. A The expression of IGF2BP3 at protein level in SGC7901 and BCG823 cells after circARID1A knockdown. B The expression of IGF2BP3 at RNA level in SGC7901 and BCG823 cells after circARID1A knockdown. C The expression of circARID1A in BGC823 or AGS cells after IGF2BP3 knockdown or overexpression. The *P*-values were calculated using the two-tailed Student’s t test. **Fig. S8** The interference efficiency of circARID1A in SGC7901 and BGC823 cells. A The expression of ARID1A in SGC7901 and BGC823 cells treated with si-circARID1A. B The expression of circARID1A in SGC7901 and BGC823 cells treated with si-circARID1A. The *P*-values were calculated using the two-tailed Student’s t test. *P < 0.05. **Fig. S9** The overexpression of circARID1A in GC cells by using different vectors. A The plasmid profile of pLC5-ciR and overexpression of pLC5-ciR-circARID1A in SGC7901 and BGC823 cells. B The plasmid profile of plv-ciR and overexpression of plv-ciR-circARID1A in SGC7901 and BGC823 cells. C The plasmid profile of pcDNA3.1(+) CircRNA Mini Vector and overexpression of pcDNA3.1-circARID1A in SGC7901 and BGC823 cells. D The overexpression of pLC5-ciR-circPDHK1 in SGC7901 and BGC823 cells. **Fig. S10** The expression of SLC7A5 protein after IGF2BP3 overexpression in SGC7901 and BGC823 cells. **Fig. S11** The expression of SLC7A5 in GC tissues and cell lines and correlation with IGF2BP3 in GC. A The expression of SLC7A5 mRNA in STAD of TCGA database. B The expression of IGF2BP3 in GC cell lines or normal gastric epithelial cells. C Correlation between SLC7A5 mRNA and IGF2BP3 mRNA in STAD of TCGA database. **Fig. S12** The effect of circARID1A knockdown on HGC-27 proliferation. A The viability of HGC-27 cells after circARID1A knockdown. B The plate colony formation of HGC-27 cells after circARID1A knockdown. C The proliferation of HGC-27 after circARID1A knockdown. The *P*-values were calculated using the two-tailed Student’s t test. *P < 0.05.

## Data Availability

All data in our study are available from the corresponding author upon reasonable request.

## Abbreviations

GC: Gastric cancer
circRNAs: Circular RNAs
IGF2BP3: Insulin like growth factor II mRNA binding protein 3
RNA-FISH: RNA fluorescence in situ hybridization
ISH: In situ hybridization
RBP: RNA binding protein
RRM: RNA recognition motifs
KH: HnRNP K homology domains
SLC7A5: Solute carrier family 7 member 5
CCK-8: cell counting Kit-8
EdU: 5-Ethynyl-20-deoxyuridine
RIP: RNA-binding protein immunoprecipitation
TMA: Tissue microarray
IHC: Immunohistochemistry
qRT-PCR: Quantitative real time -polymerase chain reaction
MOI: Multiplicity of infection
