# Supplementary material for: CircARID1A binds to IGF2BP3 in gastric cancer and promotes cancer proliferation by forming a circARID1A-IGF2BP3-SLC7A5 RNA–protein ternary complex
Source: J Exp Clin Cancer Res. 2022 Aug 19;41:251. doi: 10.1186/s13046-022-02466-3 (PMC9389715; doi:10.1186/s13046-022-02466-3)
Supplement: Supplementary file 2 — Additional file 2: Fig. S1 IGF2BP3 expression in GC tissues or cell lines. A Heat map of RBPs dysregulated in STAD of TCGA database. B Representative IHC images of IGF2BP3 expression in normal gastric mucosa or GC tissues from HPA database. C The expression of IGF2BP3 in normal gastric epithelia cell GES-1 and gastric cancer cell lines SGC7901, BGC823, AGS, and MKN74. Fig. S2 The interference efficiency and inhibiting proliferation effect of two designed IGF2BP3 siRNAs. A The interference efficiency of si-IGF2BP3-1 and si-IGF2BP3-2 in SGC7901 and BGC823 cells. B The viability of SGC7901 and BGC823 cells transfected with si-IGF2BP3-1 and si-IGF2BP3-2. C The proliferation of SGC7901 and BGC823 cells transfected with si-IGF2BP3-1 and si-IGF2BP3-2. The P-values were calculated using the two-tailed Student’s t test. *P < 0.05. Fig. S3 The expression of IGF2BP3 in SGC7901 and BGC823 cells after IGF2BP3 knockdown or overexpression. A The expression of IGF2BP3 in SGC7901 and BGC823 cells after IGF2BP3 knockdown by siRNA. B The expression of IGF2BP3 in SGC7901 and BGC823 cells with IGF2BP3 stable overexpression. Fig. S4 Screening of circRNAs that binding to IGF2BP3 protein in GC. A Heat map of circRNAs binding to IGF2BP3 in SGC7901 cells evaluated by RIP-seq. B The expression of circELK4 in GC tissues (GC) and adjacent tissues (NC). The P-value was calculated using the two-tailed Student’s t test. Fig. S5 CircARID1A sequencing results of GC tissues and the adjacent tissues. A CircARID1A sequencing results of GC tissue and the adjacent tissue from patient 1. B CircARID1A sequencing results of GC tissue and the adjacent tissue from patient 2. Fig. S6 Validation of the binding between circARID1A and IGF2BP3 proteins in BGC823 cells. A RIP analysis of circARID1A enrichment pull-downs by GFP in BGC823 cells overexpressing EGFP-tagged IGF2BP3. B-C RIP analyses of circARID1A enrichment pull-downs by IGF2BP3 in BGC823 cells following (B) IGF2BP3 or (C) circARID1A knockdowns. D Enric [file 13046_2022_2466_MOESM2_ESM.docx]

**Supplementary Figures**


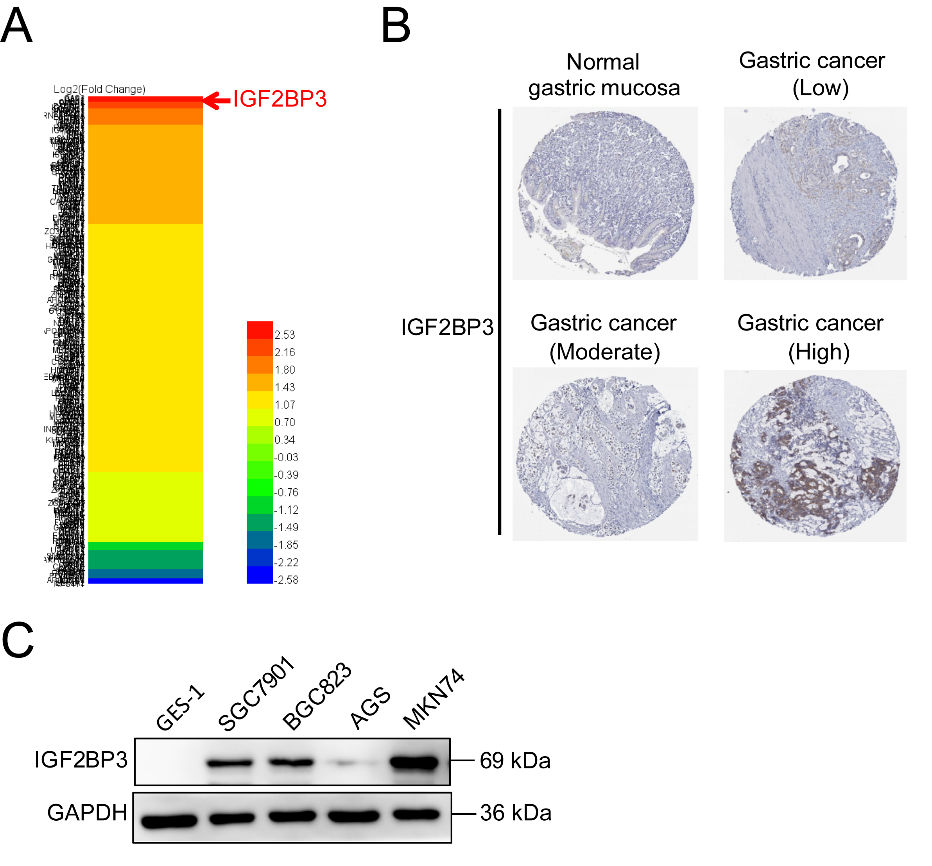


**Fig. S1** IGF2BP3 expression in GC tissues or cell lines. **A** Heat map of RBPs dysregulated in STAD of TCGA database. **B** Representative IHC images of IGF2BP3 expression in normal gastric mucosa or GC tissues from HPA database. **C** The expression of IGF2BP3 in normal gastric epithelia cell GES-1 and gastric cancer cell lines SGC7901, BGC823, AGS, and MKN74.


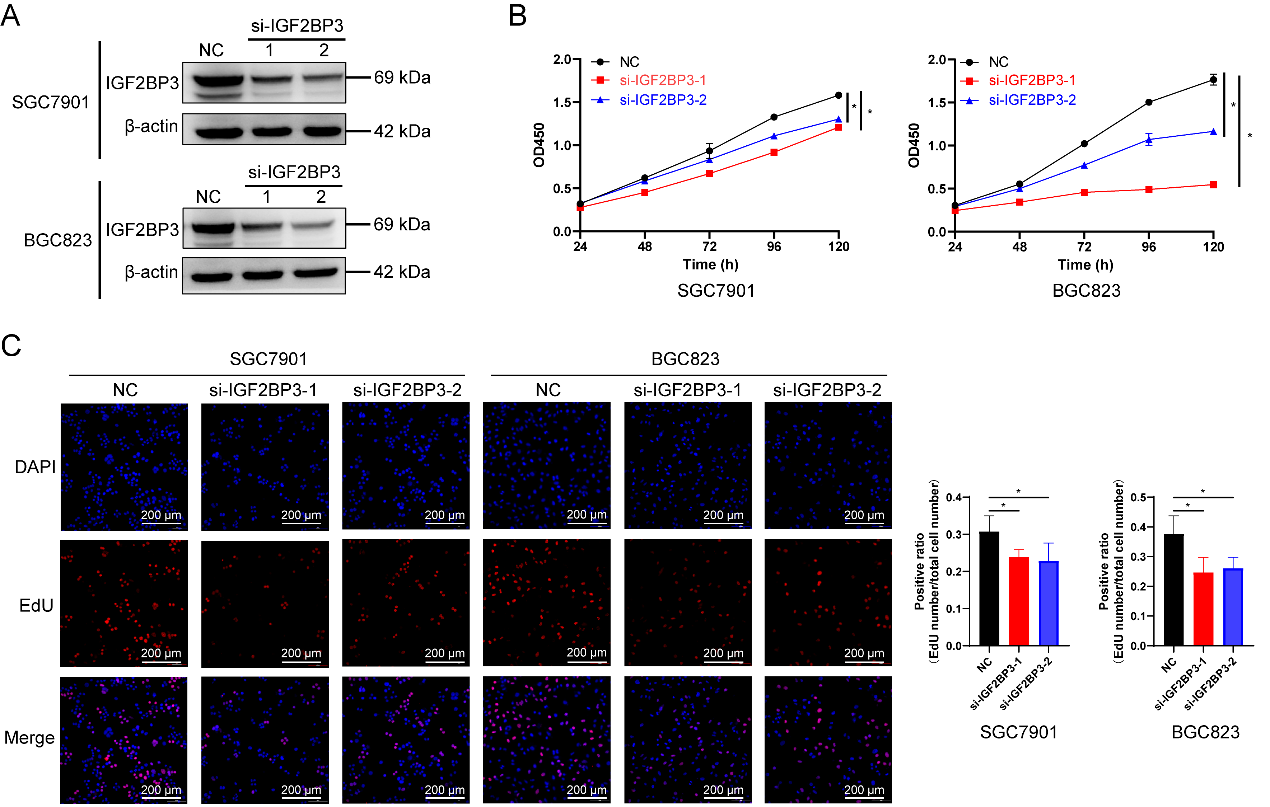


**Fig. S2** The interference efficiency and inhibiting proliferation effect of two designed IGF2BP3 siRNAs. **A** The interference efficiency of si-IGF2BP3-1 and si-IGF2BP3-2 in SGC7901 and BGC823 cells. **B** The viability of SGC7901 and BGC823 cells transfected with si-IGF2BP3-1 and si-IGF2BP3-2. **C** The proliferation of SGC7901 and BGC823 cells transfected with si-IGF2BP3-1 and si-IGF2BP3-2. The *P*-values were calculated using the two-tailed Student’s t test. **P* < 0.05.


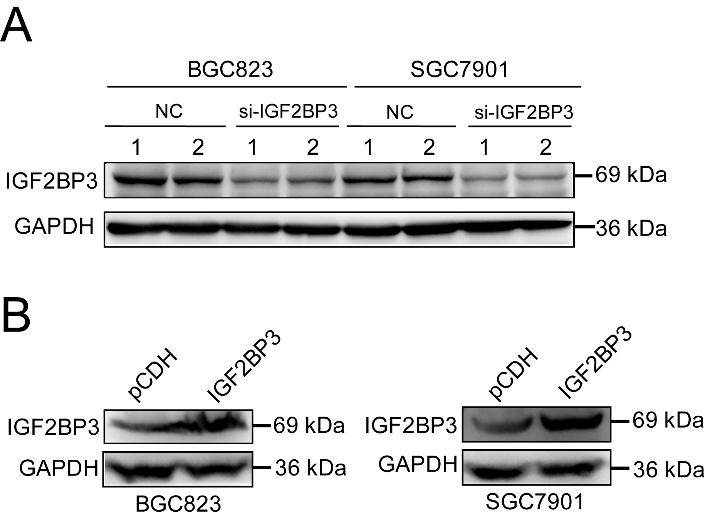


**Fig. S3** The expression of IGF2BP3 in SGC7901 and BGC823 cells after IGF2BP3 knockdown or overexpression. **A** The expression of IGF2BP3 in SGC7901 and BGC823 cells after IGF2BP3 knockdown by siRNA. **B** The expression of IGF2BP3 in SGC7901 and BGC823 cells with IGF2BP3 stable overexpression.


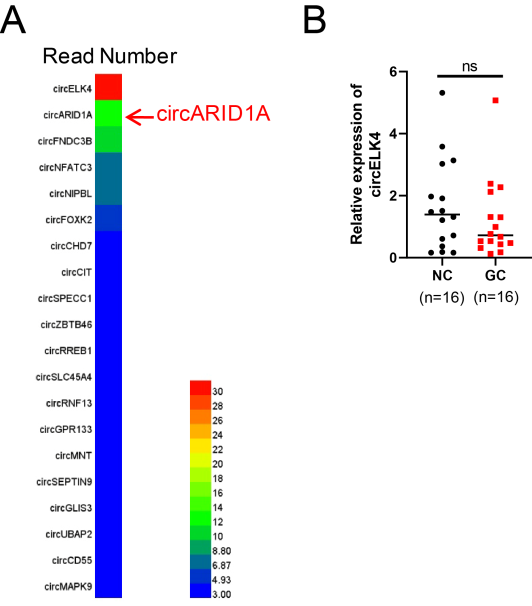


**Fig. S4** Screening of circRNAs that binding to IGF2BP3 protein in GC. **A** Heat map of circRNAs binding to IGF2BP3 in SGC7901 cells evaluated by RIP-seq. **B** The expression of circELK4 in GC tissues (GC) and adjacent tissues (NC). The *P*-value was calculated using the two-tailed Student’s t test.


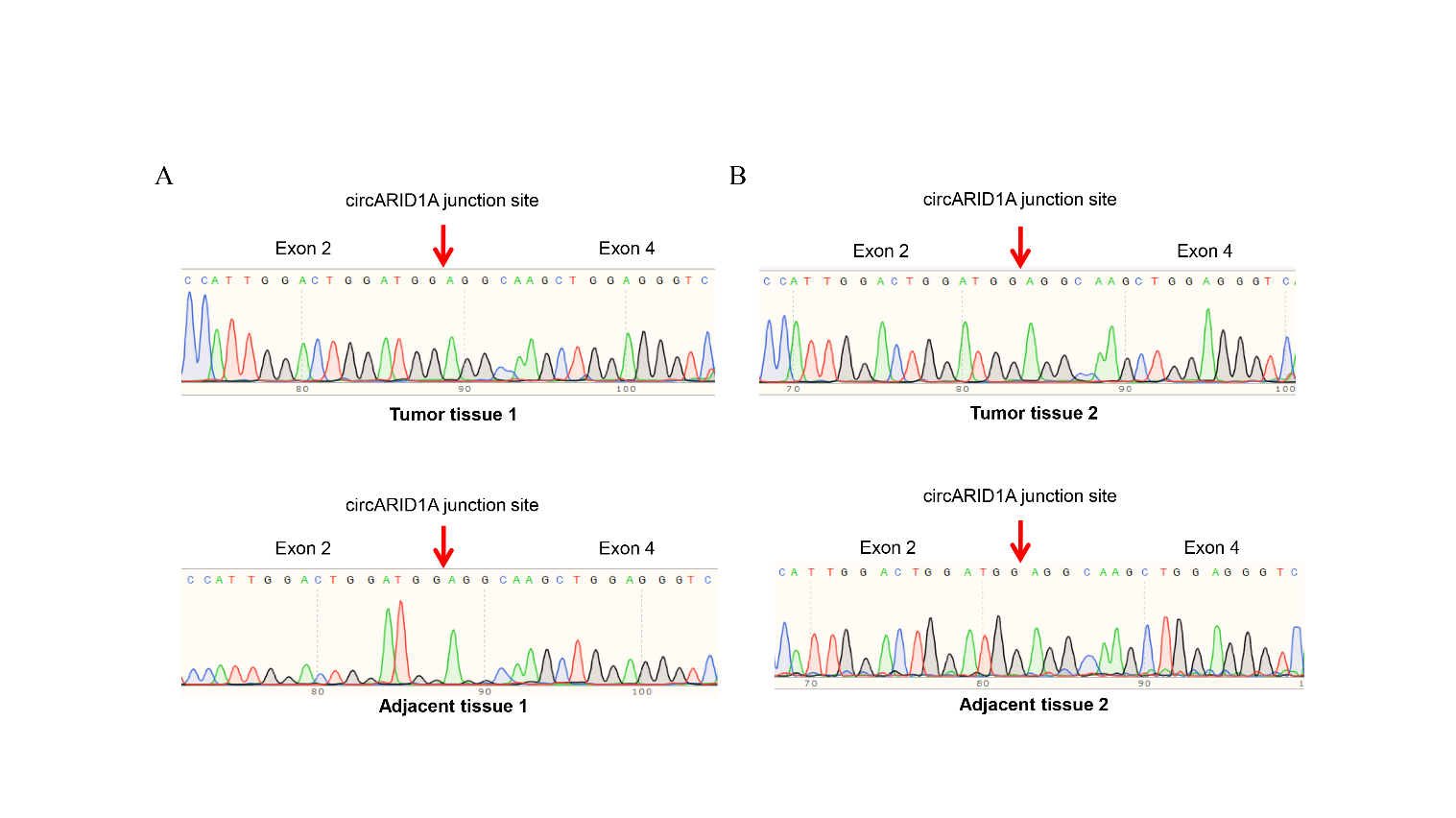


**Fig. S5** CircARID1A sequencing results of GC tissues and the adjacent tissues. **A** CircARID1A sequencing results of GC tissue and the adjacent tissue from patient 1. **B** CircARID1A sequencing results of GC tissue and the adjacent tissue from patient 2.


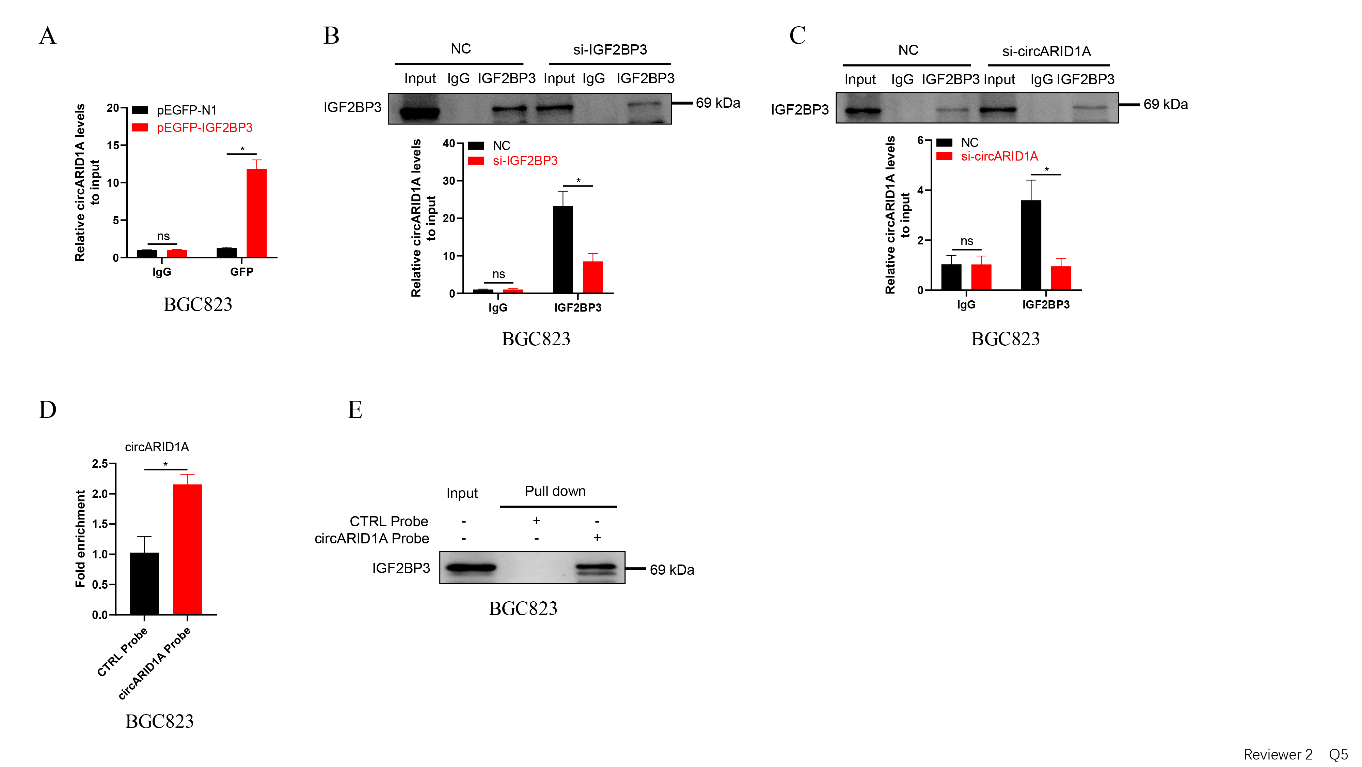


**Fig. S6** Validation of the binding between circARID1A and IGF2BP3 proteins in BGC823 cells. **A** RIP analysis of circARID1A enrichment pull-downs by GFP in BGC823 cells overexpressing GFP-tagged IGF2BP3. **B-C** RIP analyses of circARID1A enrichment pull-downs by IGF2BP3 in BGC823 cells following (B) IGF2BP3 or (C) circARID1A knockdowns. **D** Enrichment efficiency of biotin tagged circARID1A probes assessed by RNA pull-downs. **E** Western blot validation of interaction of circARID1A and IGF2BP3 by RNA pull-down in BGC823 cells. The *P*-values were calculated using the two-tailed Student’s t test. **P* < 0.05.


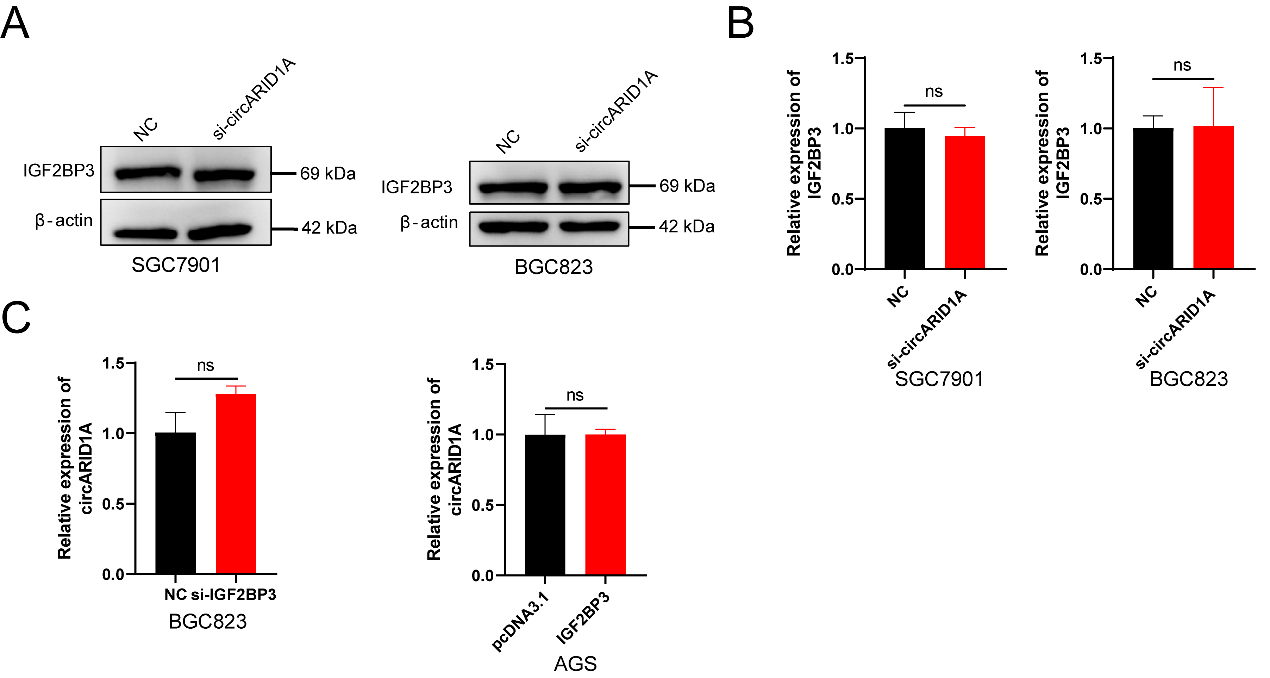


**Fig. S7** Regulation of circARID1A and IGF2BP3 to each other in GC cells. **A** The expression of IGF2BP3 at protein level in SGC7901 and BCG823 cells after circARID1A knockdown. **B** The expression of IGF2BP3 at RNA level in SGC7901 and BCG823 cells after circARID1A knockdown. **C** The expression of circARID1A in BGC823 or AGS cells after IGF2BP3 knockdown or overexpression. The *P*-values were calculated using the two-tailed Student’s t test.


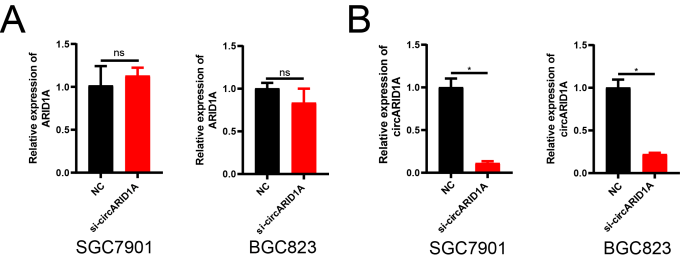


**Fig. S8** The interference efficiency of circARID1A in SGC7901 and BGC823 cells. **A** The expression of ARID1A in SGC7901 and BGC823 cells treated with si-circARID1A. **B** The expression of circARID1A in SGC7901 and BGC823 cells treated with si-circARID1A. The *P*-values were calculated using the two-tailed Student’s t test. **P* < 0.05.


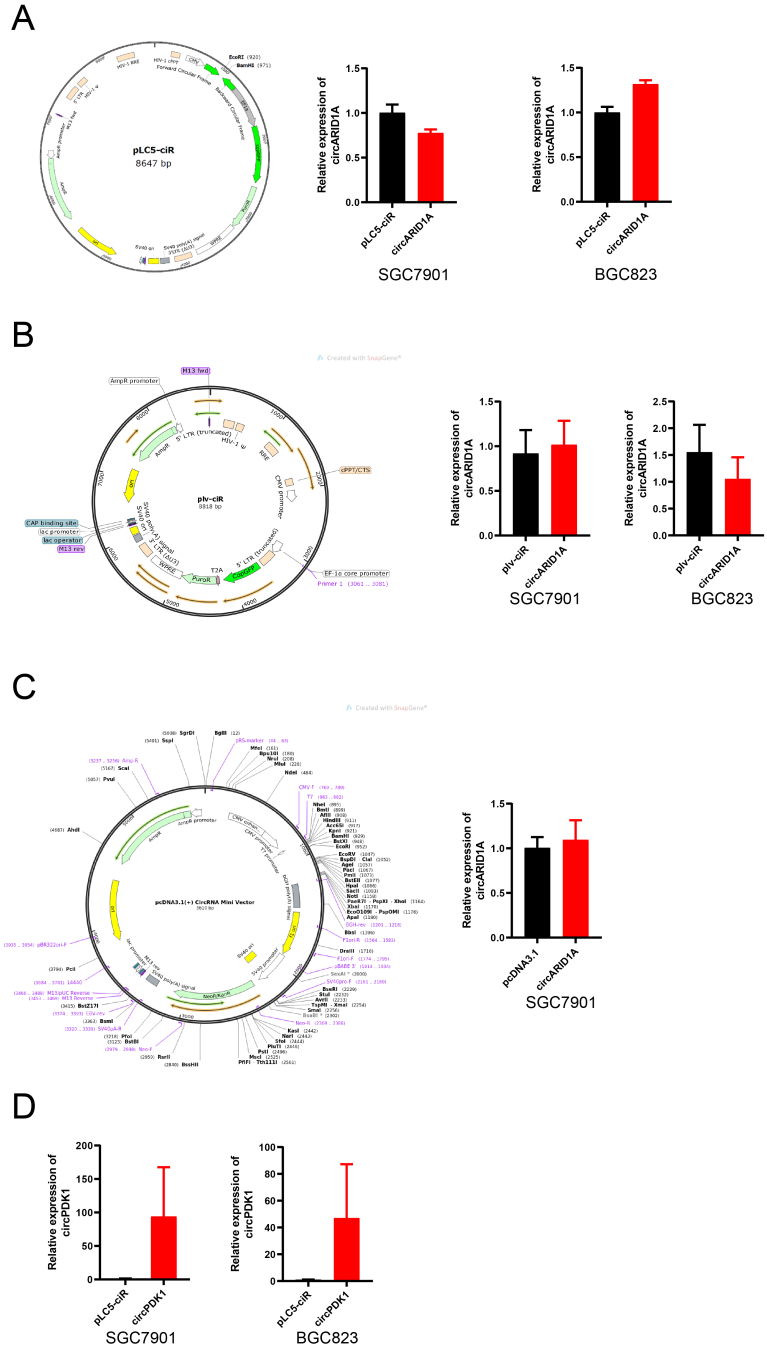


**Fig. S9** The overexpression of circARID1A in GC cells by using different vectors. (A) The plasmid profile of pLC5-ciR and overexpression of pLC5-ciR-circARID1A in SGC7901 and BGC823 cells; (B) The plasmid profile of plv-ciR and overexpression of plv-ciR-circARID1A in SGC7901 and BGC823 cells; (C) The plasmid profile of pcDNA3.1(+) CircRNA Mini Vector and overexpression of pcDNA3.1-circARID1A in SGC7901 and BGC823 cells; (D) The overexpression of pLC5-ciR-circPDHK1 in SGC7901 and BGC823 cells.


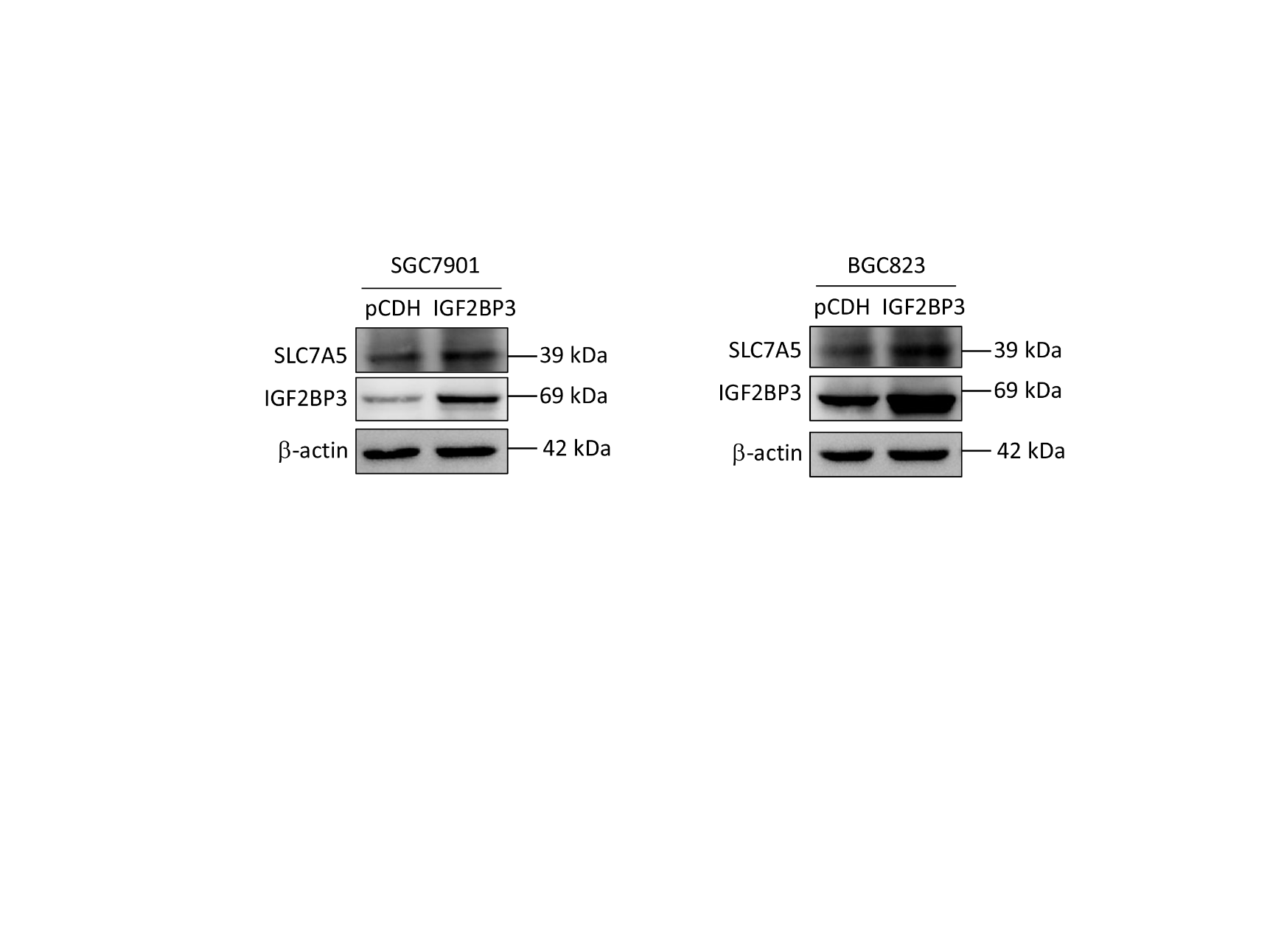


**Fig. S10** The expression of SLC7A5 protein after IGF2BP3 overexpression in SGC7901 and BGC823 cells.


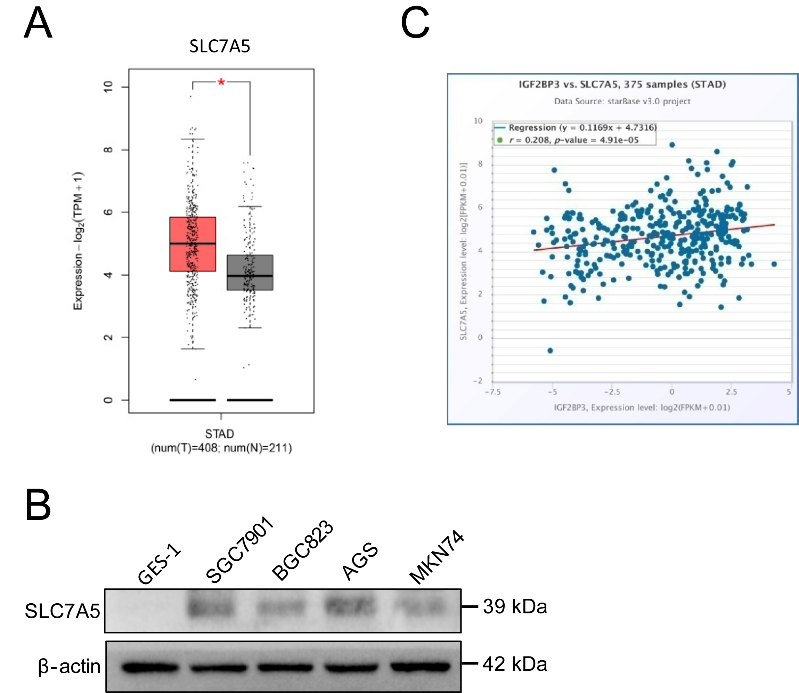


**Fig. S11** The expression of SLC7A5 in GC tissues and cell lines and correlation with IGF2BP3 in GC. (A) The expression of SLC7A5 mRNA in STAD of TCGA database. (B) The expression of IGF2BP3 in GC cell lines or normal gastric epithelial cells. (C) Correlation between SLC7A5 mRNA and IGF2BP3 mRNA in STAD of TCGA database.


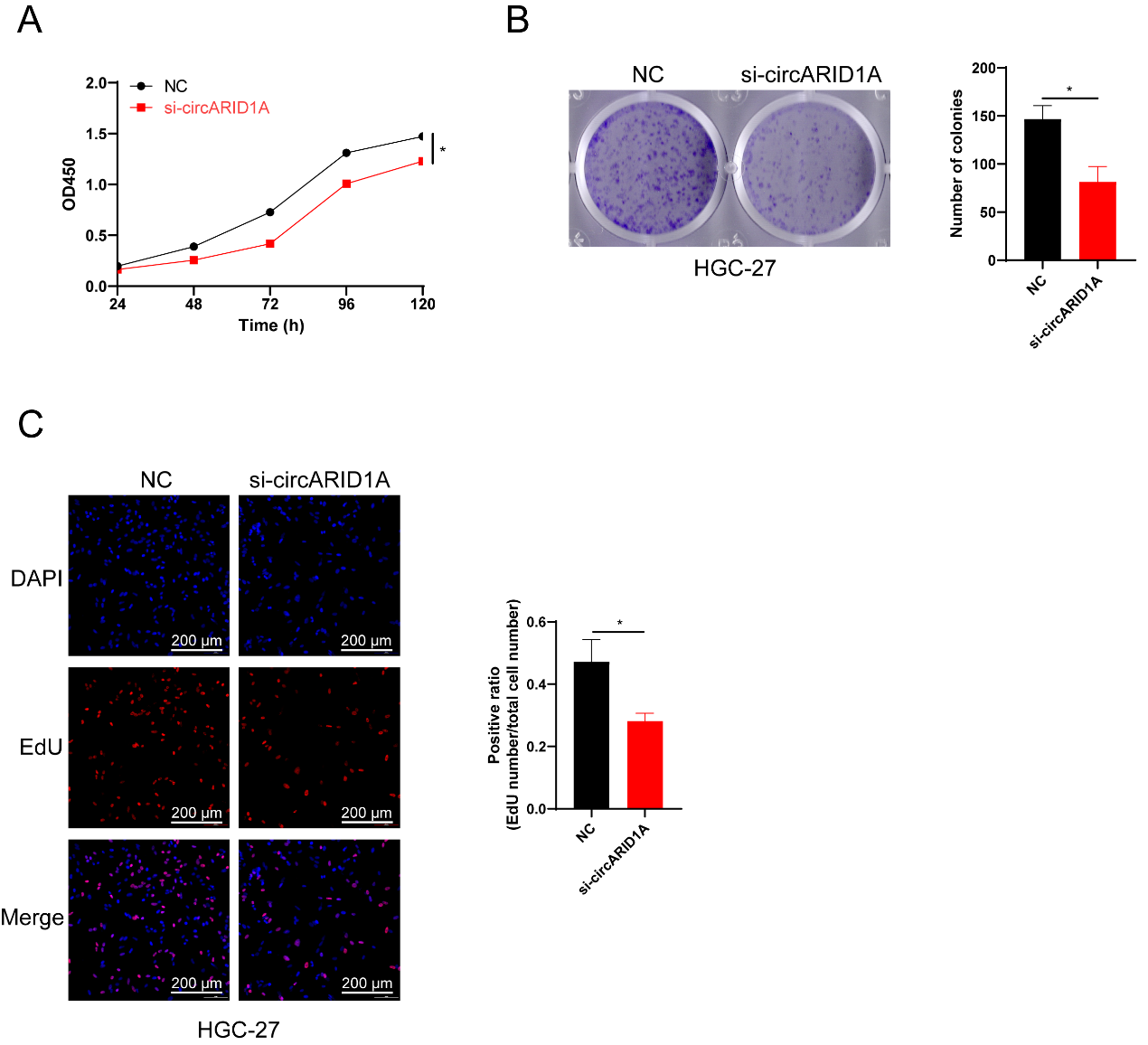


**Fig. S12** The effect of circARID1A knockdown on HGC-27 proliferation. (A) The viability of HGC-27 cells after circARID1A knockdown. (B) The plate colony formation of HGC-27 cells after circARID1A knockdown. (C) The proliferation of HGC-27 after circARID1A knockdown. The *P*-values were calculated using the two-tailed Student’s t test. **P* < 0.05.
